# Sulfur-Containing Compounds from Endophytic Fungi: Sources, Structures and Bioactivities

**DOI:** 10.3390/jof8060628

**Published:** 2022-06-13

**Authors:** Yaqin Fan, Zhiheng Ma, Yan Zhang, Yufei Wang, Yousong Ding, Cong Wang, Shugeng Cao

**Affiliations:** 1 Shandong Provincial Key Laboratory of Applied Mycology, School of Life Sciences, Qingdao Agricultural University, Qingdao 266109, China; fanyaqin@qau.edu.cn (Y.F.); 20202206023@stu.qau.edu.cn (Z.M.); 20212206060@stu.qau.edu.cn (Y.Z.); 2 Key Laboratory of Chemistry and Engineering of Forest Products, State Ethnic Affairs Commission, Guangxi Collaborative Innovation Center for Chemistry and Engineering of Forest Products, School of Chemistry and Chemical Engineering, Guangxi Minzu University, Nanning 530006, China; 171416020323@stu.haust.edu.cn; 3 Department of Medicinal Chemistry, Center for Natural Products, Drug Discovery and Development, College of Pharmacy, University of Florida, Gainesville, FL 32610, USA; yding@cop.ufl.edu; 4 Department of Pharmaceutical Sciences, Daniel K. Inouye College of Pharmacy, University of Hawai’i at Hilo, 200 W. Kawili St., Hilo, HI 96720, USA

**Keywords:** sulfur, plant endophyte, endophytic fungi

## Abstract

Endophytic fungi have attracted increasing attention as an under-explored source for the discovery and development of structurally and functionally diverse secondary metabolites. These microorganisms colonize their hosts, primarily plants, and demonstrate diverse ecological distribution. Among endophytic fungal natural products, sulfur-containing compounds feature one or more sulfur atoms and possess a range of bioactivities, e.g., cytotoxicity and antimicrobial activities. These natural products mainly belong to the classes of polyketides, nonribosomal peptides, terpenoids, and hybrids. Here, we reviewed the fungal producers, plant sources, chemical structures, and bioactivities of 143 new sulfur-containing compounds that were reported from 1985 to March 2022.

## 1. Introduction

Sulfur is one of the prime elements on Earth and the eighth most abundant element in the human body. It is a group 6A (or VIA) member of the periodic table, with a larger atomic size and a weaker electronegativity than oxygen. Sulfur has unique characteristics, such as five different oxidation states, and sulfur-containing molecules often participate in biological redox reactions and electron transfer processes. Notably, two essential amino acids, l-methionine and l-cysteine, both contain a sulfur atom, further highlighting the importance and indispensability of sulfur in biology [1]. Indeed, one fifth (20%) of the FDA-approved drugs contain at least one sulfur atom. These sulfur-containing drugs have different structure skeletons such as sulfonamides, β-lactams, thioethers, thiazoles, thiophenes, phenothiazines, sulfoxides, S=C and S=P structures, thionucleotides, sulfones, sulfates and macrocyclic disulfides. Of note, many sulfur-containing drugs are natural products or their derivatives (i.e., rosuvastatin, ecteinascidin 743 and ixabepilone) [2].

Fungi are a major group of microorganisms that produce a broad array of compounds with novel structures and unique bioactivities. One type of fungi colonizes the intercellular and/or intracellular regions of healthy plant tissues at a particular time and has no interference with and causes no pathogenic symptoms to the host [3]. These endophytic microorganisms are an important but less-explored source for the discovery of structurally novel natural products in drug research. This paper reviews new sulfur-containing compounds isolated from endophytic fungi since 1985 (Table 1). Based on their major chemical features, these compounds will be categorized into peptides, disulfides, polyketides, hybrids and terpenoids. The fungal strains that producing sulfur-containing compounds, host plants, structure uniqueness and biological activities of these compounds will be discussed (Table 1). 

## 2. Peptides

### 2.1. Sulfide (R-S-R′)

**Figure 1:**
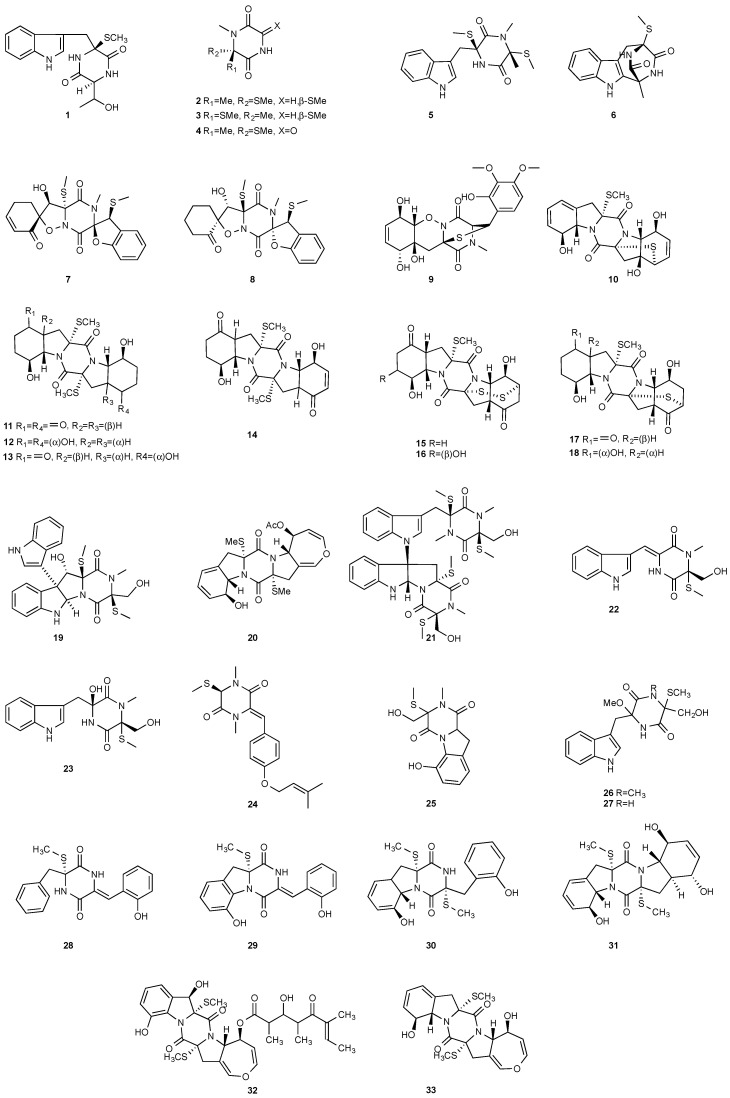
Structures of compounds **1**–**33**.

A rare diketopiperazine bionectin D (**1**) (Figure 1) was obtained from a fungal strain *Bionectria* sp. Y1085 that was isolated from the plant *Huperzia serrata*. Bionectin D (**1**) consists of a tryptophan and a threonine moiety, and the α-carbon of its tryptophan moiety carries a single methylthio substitution. Compound **1** exhibited antibacterial activity against *Staphylococcus aureus*, *Escherichia coli*, and *Salmonella typhimurium* ATCC 6539 with the same minimal inhibitory concentration (MIC) of 25 μg/mL [4]. Lasiodiplines A-C (**2**–**4**) and E-F (**5**–**6**) are new sulfureous diketopiperazines that were produced by *Lasiodiplodia pseudotheobromae* F2 isolated from the apparently normal flower of *Illigera rhodantha*. The structure elucidation of these compounds was accomplished using a combination of spectroscopic and computational approaches, and the structure of **2** was further confirmed in conjunction with low-temperature (100 K) single-crystal X-ray diffraction. Lasiodiplines E (**5**) displayed antibacterial activity against *Veillonella parvula*, *Actinmyces israelili*, *Streptococcus* sp., *Bacteroides vulgates* and *Peptostreptococcus* sp. with the MIC values of 0.25, 32.0, 0.12, 0.12 and 0.12 μg/mL, respectively [5].

Botryosulfuranols A and B (**7**–**8**), two spirocyclic thiodiketopiperazines, were purified from *Botryosphaeria mamani*. The fungal strain was isolated from the fresh leaves of *Bixa orellana* L. (Bixaceae) collected in Peru. These two unique compounds, each of which contains two spiro centers, were derived from two l-phenylalanines with two methylthio substitutions at the α-carbon and β-carbon of the two building blocks, respectively. Botryosulfuranols A (**7**) was active against four cancer cell lines (HT-29, HepG2, Caco-2, HeLa) with IC_50_ values of 8.0, 11.4, 18.2, 23.5 and 9.3 μM, respectively. Botryosulfuranols B (**8**) was active against three cancer cell lines (HT-29, HepG2, HeLa) with the IC_50_ values of 63.2, 56.1, 61.2, 49.9 and 64.7 μM, respectively [6]. Outovirin A (**9**) was a thiodiketopiperazine derived from two molecules of l-phenylalanine. It was produced by *Penicillium raciborskii*, an endophytic fungus isolated from *Rhododendron tomentosum* [7]. Compound **9** contains a nitrogen-oxygen bond in the oxazinane ring between diketopiperazine and conduritol-like rings, and it has a sulfide bridge between the α- and β-carbons rather than the typical α−α bridging. Nine new thiodiketopiperazines, epicoccin I (**10**), ent-epicoccin G (**11**), and epicoccins J-P (**12**–**18**), have been isolated from the endophytic fungus *Epicoccum nigrum*. Compounds **10**, **17**, and **18** all have a sulfide bridge between the α-carbon and the 2′/3′-position of the reduced benzene ring. Ent-epicoccin G (**11**) and epicoccins M (**15**) showed potent in vitro activities against the release of β-glucuronidase in rat polymorphonuclear leukocytes induced by the platelet-activating factor, with IC_50_ values of 3.07 and 4.16 µM, respectively [8]. 

*Tilachlidium* sp. (CANU-T988) isolated from a decaying wood sample was reported to produce T988 B (**19**). Compound **19** has an unusual dimerized indole moiety with a 3-3 linkage, and it displayed potent cytotoxicity against P388 leukemia cells with an IC_50_ of 2.18 µM [9]. Bisdethiobis(methylsulfanyl)apoaranotin (**20**) was produced by *Aspergillus terreus* BCC 4651, which was isolated from a tree hole in Nam Nao National Park, Thailand. Compound **20** was derived from two molecules of l-phenylalanine with one benzene ring being oxidized to a 4,5-dihydrooxepine ring. Compound **20** exhibited weak antimycobacterial activity [10]. Chaetocochin G (**21**), oidioperazine E (**22**), and chetoseminudin E (**23**) were obtained from *Chaetomium* sp 88194, which was isolated from *Cymbidium goeringii*, a plant native to China, Japan and Korea. Chaetocochin G (**21**) is a dimer of serine-tryptophan diketopiperazines. Its structure including the absolute configuration was established by spectroscopic data interpretation and single-crystal X-ray diffraction analysis. Chaetocochin G (**21**) showed cytotoxicity against MCF-7 [11]. Fusaperazine E (**24**) and colletopiperazine (**25**) were obtained from *Penicillium crustosum* and *Colletotrichum gloeosporioides*, respectively. Both strains were isolated from *Viguiera robusta* Gardn. (Asteraceae). [12].

Chetoseminudin F and G (**26**–**27**) were purified from *Chaetomium* sp. SYP-F7950, which was isolated from the root of *Panax notoginseng* collected from Wenshan, Yunnan, P. R. China. Chetoseminudin F (**26**) displayed cytotoxicity against MDA-MB-231 with an IC_50_ of 26.49 μM [13]. Four thiodiketopiperazines penicibrocazines F–I (**28**–**31**) were purified from *Penicillium brocae* MA-231, which was isolated from the fresh tissue of the marine mangrove plant *Avicennia marina* collected at Hainan Island, P. R. China. Penicibrocazines H (**30**) displayed activity against *V. harveyi*, *E. coli*, *A. hydrophilia* and *V. parahaemolyticus* with MICs of 16.0, 16.0, 32.0, and 16.0 μg/mL, respectively. Penicibrocazines I (**31**) displayed activity against *V. harveyi* with an MIC of 32.0 μg/mL [14].

Two new compounds 6-octenoic acid, 3-hydroxy-2,4,6-trimethyl-5-oxo-, (5S,5aS,7aR,8R,14aR)-5,5a,7a,8,14a,15-hexahydro-8,12-dihydroxy-7a,14a-bis(methylthio)-7,14-dioxo-7H,14H-oxepino[3″,4″:4′,5′]pyrrolo[1′,2′:4,5]pyrazino[1,2-a]indol-5-yl ester (6E) (**32**) and bisdethiobis(methylthio)deacetylapoaranotin (**33**) were purified from the seed fungus *Menisporopsis theobromae* BCC3975. Compound **32** is a hybrid of diketopiperazine and polyketide. Both compounds showed antimycobacterial activity with MICs of 1.24 and 7.14 µM, respectively. Compound **32** displayed cytotoxicity against NCI-H187 cell line and antimalarial activity with IC_50_ of 20.3 and 2.95 μM, respectively [15].

**Figure 2:**
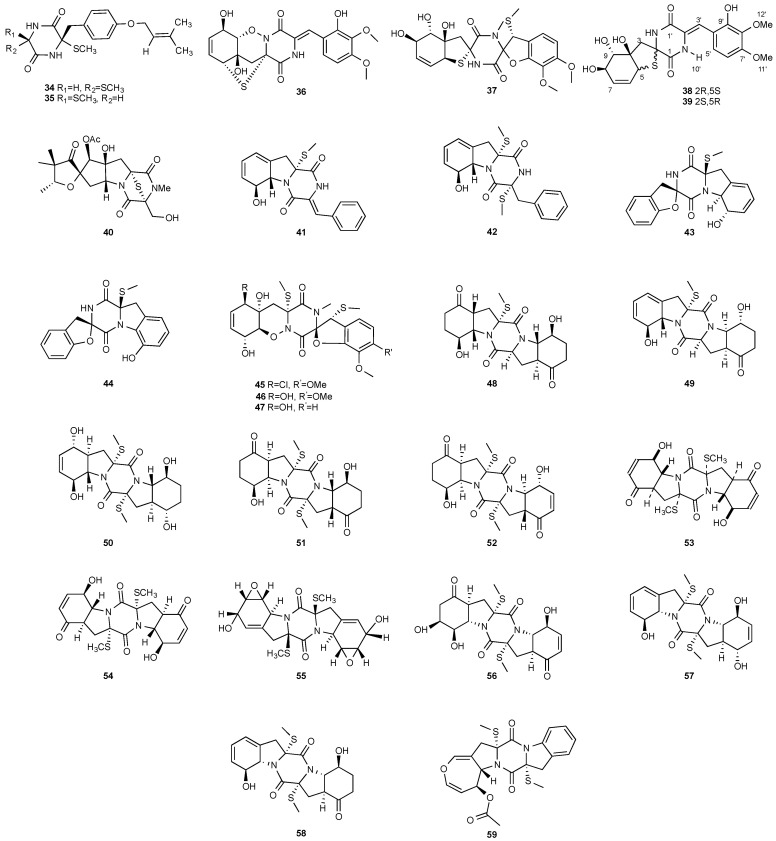
Structures of compounds **34**–**59**.

Two new compounds, Sch 54794 (**34**) and Sch 54796 (**35**) (Figure 2), were separated from the fermentation culture of *ToJypocJadium* sp. The microorganism *ToJypocJadium* sp. was isolated from dead twigs from a *Quercus virginiana* Miller, an old live oak tree in the state of Tamalupas, Mexico. The structures of Sch 54794 (**34**) and Sch 54796 (**35**) were determined as *cis* and *trans* isomers in the spectroscopic analysis. The *trans* isomer, which was similar to other diketopiperazines reported as platelet-activating factor (PAF) inhibitors in the literature, displayed weak inhibitory activity in PAF assay with an IC_50_ of 50 μM. However, the *cis* isomer appeared inactive (IC_50_ > 100 μM) [16].

Four new dioxopiperazine alkaloids, penispirozines A−D (**36**–**39**), were produced by *Penicillium janthinellum* HDN13-309, which was isolated from the root of the mangrove plant *Sonneratia caseolaris*. Penispirozine A (**36**) contains an unusual pyrazino[1,2]oxazadecaline coupled with a thiophane ring system, and compound **37** possesses a 6/5/6/5/6 pentacyclic ring system with two rare spirocyclic centers. Penispirozines C (**38**) and penispirozines D (**39**) increased the expression of superoxide dismutase 2 (SOD2) and heme oxygenase-1 (HO-1) at 10 μM [17]. A fermentation broth of *Phoma lingam* isolate Leroy obtained from rapeseeds generated a new compound sirodesmin H (**40**) [18]. The octahydrocyclopenta[*b*]pyrrole moiety in **40** might be derived from l-phenylalanine, which reacted with an isoprenyl group (C_5_) to form a spiro-furanone system. Two new thiodiketopiperazines phomazines A (**41**) and B (**42**) were purified from *Phoma* sp. OUCMDZ-1847, which was isolated from the mangrove plant *Kandelia candel* at Wenchang, Hainan, P. R. China. Compound **42** displayed inhibitory activity against MGC-803 cells with an IC_50_ of 8.5 μM [19].

Two new pentacyclic diketopiperazines spirobrocazines A (**43**) and B (**44**) were obtained from *Penicillium brocae* MA-231, which was derived from the marine mangrove plant *Avicennia marina* [20]. Compound **43** exhibited moderate antibacterial activities against *Escherichia coli*, *S. aureus* and *Vibrio harveyi* with MIC values of 32.0, 16.0 and 64.0 μg/mL, respectively. Three new epipolythiodioxopiperazines, penicisulfuranols D–F (**45**–**47**), were isolated from a marine mangrove plant, *Sonneratia caseolaris*-derived *Penicillium janthinellum* HDN13-309 [21]. The piperazine-2,5-dione core in each of these compounds (**45**–**47**) was flanked by a 1,2-oxazadecaline moiety and a *spiro*-benzofuran ring. Compounds **45**–**47** were tested inactive against HeLa and HL-60 cell lines. Five pentacyclic diketopiperazines, penicibrocazines A–E (**48**–**52**), were obtained from *Penicillium brocae* MA-231, a fungus obtained from the fresh tissue of the marine mangrove plant *Avicennia marina*. In the antimicrobial screening, penicibrocazine B (**49**), penicibrocazine C (**50**) and penicibrocazine D (**51**) showed activity against *Staphylococcus aureus*, with MIC values of 32.0, 0.25, 8.0 μg/mL, respectively, which are comparable with that of the positive control, chloromycetin (MIC = 4.0 μg/mL). Penicibrocazines C (**50**) also showed activity against *Micrococcus luteus* with an MIC of 0.25 μg/mL, which is stronger than that of the positive control, chloromycetin (MIC = 2.0 μg/mL). Moreover, penicibrocazines B (**49**) and D (**51**) exhibited activity against the plant pathogen *Gaeumannomyces graminis* with MIC values of 0.25 and 8.0 μg/mL, respectively, while the positive control amphotericin B has an MIC of 16.0 μg/mL [22]. 

The chemical investigation of a culture of *Exserohilum holmii*, a pathogenic fungus of the weedy plant *Dactyloctenium aegyptium*, yielded two linearly fused pentacyclic diketopiperazines exserohilone (**53**) and 9,10-Dihydroexserohilon (**54**) [23]. The fermentation of *Nigrospora sphaerica*, which was isolated from a germinating fescue seed, on shredded wheat medium generated a novel pentacyclic diketopiperazine, epoxyexserohilone (**55**), a congener of the known phytotoxin, exserohilone [24]. The investigation of *Setosphaeria rostrata* led to the discovery of three pentacyclic diketopiperazines, rostratazines A-C (**56**–**58**). The fungal strain was isolated from the fresh leaf tissues of the medicinal plant *C. speciosus* collected from Colombo, Sri Lanka. Rostratazine B (**57**) inhibited porcine pancreatic alpha-amylase activity with an IC_50_ of 578 μM [25]. A pentacyclic diketopiperazine with a 4,5-dihydrooxepine moiety versicolor A (**59**) was isolated from *Aspergillus versicolor* 0312. The fungal strain was isolated from the stems of *Paris polyphylla var. yunnanensis* collected in Kunming, Yunnan Province, P. R. China. Compound **59** displayed cytotoxicity against the contraction of the MOLT-4 cell line with an IC_50_ of 29.6 μM [26].

### 2.2. Disulfide (R-S-S-R′) and Multisulfide (R-S_n_-S-R′, n = 3 or More)

**Figure 3:**
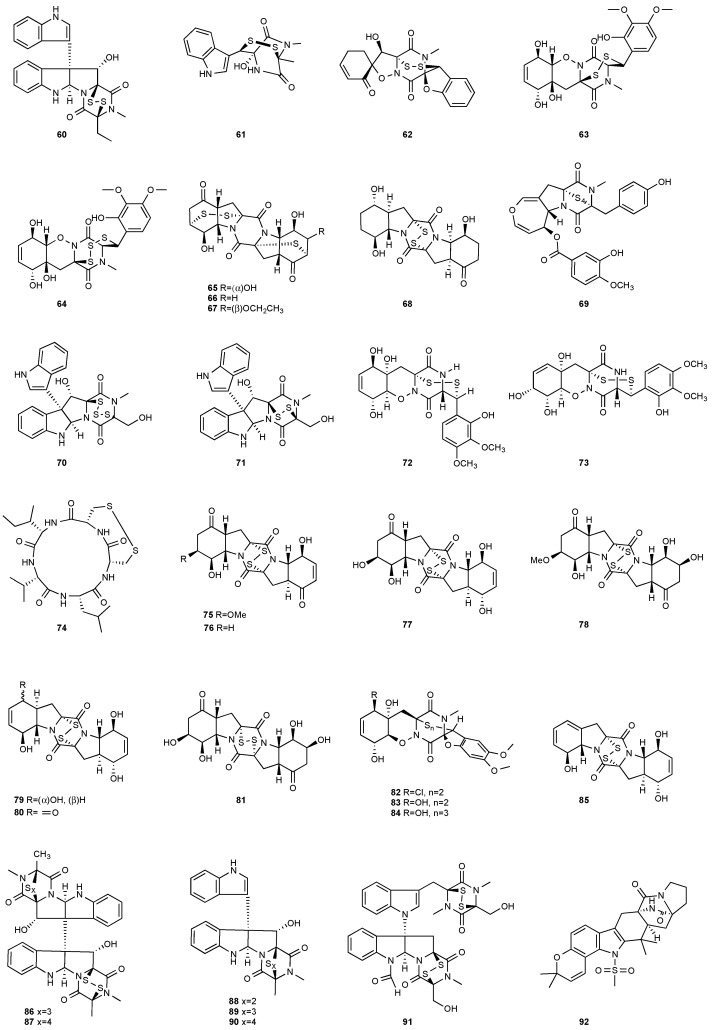
Structures of compounds **60**–**92**.

Bionectin E (**60**) (Figure 3) was obtained from *Bionectria* sp. Y1085, which was isolated from *Huperzia serrata*. Similar to compound **19** (T988 B) [9], compound **60** has an indole moiety attached to the tryptophan-derived 1,2,3,3a,8,8a-hexahydropyrrolo[2,3-*b*]indole. Interestingly, the other amino acid in the α−α′-bridged disulfide diketopiperazine is a dehydroxylated threonine. Compound **60** showed antibacterial activity against *E. coli*, *S. saureus* and *Salmonella typhimurium* with the same MIC value of 12.5 μg/mL [1]. Derived from the apparently normal flower of *Illigera rhodantha*, *Lasiodiplodia pseudotheobromae* F2 produced Lasiodipline D (**61**) [5]. The α position of the alanine moiety in compound **61** was connected to the β position of the tryptophan moiety via a disulfide bond. Botryosulfuranol C (**62**) was obtained from the same fungal strain *Botryosphaeria mamani* as compounds **7** (botryosulfuranols A) and **8** (botryosulfuranols B), but it has an α−β-bridged disulfide bond instead of the sulfide bond in **7** and **8**. Botryosulfuranol C (**62**) showed cytotoxicity against HepG2, HT29, Hela, IEC6 and Vero with IC_50_ values ranging from 15.9 to 115.7 µΜ [6].

Two new epithiodiketopiperazine natural products, outovirins B (**63**) and C (**64**), resembling the antifungal natural product gliovirin have been identified in an extract of *Penicillium raciborskii*, an endophytic fungus isolated from *Rhododendron tomentosum* [7]. Compounds **63** and **64** were almost identical to compound **9** (outovirins A) except for an α−β-bridged disulfide and a trisulfide bond in compounds **63** and **64**, respectively. Compound **64** inhibited the growth of all tested fungal isolates (*Fusarium oxysporum*, *Botrytis cinerea*, and *Verticillium dahliae*) at a low concentration of 0.38 mM (207 μg/mL), but a more significant growth inhibition was observed at 0.76 mM (413 μg/mL). Compound **64** was the most active against *Botrytis cinerea* (57% inhibition) and slightly less effective against *Verticillium dahliae* (45% inhibition). Four new pentacyclic thiodiketopiperazines, epicoccins Q-T (**65**–**68**), were discovered from the same fungal strain, *Epicoccum nigrum*, as compounds **10**–**18**. Epicoccins S (**67**) showed activity against the release of β-glucuronidase with an IC_50_ of 4.95 µM [8].

Secoemestrin D (**69**), a new epitetrathiodioxopiperizine, was obtained from *Emericella* sp. AST0036, a fungal endophyte of *Astragalus lentiginosus*. Compound **69** contains an α−α-bridged tetrasulfide bond. A benzoic acid moiety was attached to the 4,5-dihydrooxepine ring. Secoemestrin D (**69**) exhibited potent cytotoxic activity against a panel of seven cancer cell lines with IC_50_ values ranging from 0.06 to 0.24 μM [27]. *Tilachlidium* sp. (CANU-T988), a fungal strain isolated from a decaying wood sample collected in Christchurch, New Zealand, produced two new thiodiketopiperazine derivatives, T988 A (**70**) and C (**71**), which have an indole ring connected to the 1,2,3,3a,8,8a-hexahydropyrrolo[2,3-*b*]indole, structurally similar to compounds **19** and **60**. Compound **71** has an α−α-bridged disulfide bond, while compound **70** has an α−α-bridged trisulfide bond. Compounds **70** and **71** displayed cytotoxicity against P388 with IC_50_ values of 0.25 and 0.56 µM, respectively [9]. Pretrichodermamide A (**72**) was obtained from *Trichoderma* sp. BCC 5926, which was collected on a bamboo leaf from Khao Yai National Park, Nakhon Ratchasima Province, Thailand. Under alkaline conditions, compound **72** with an α−β-bridged disulfide bond underwent a rapid transformation to a stable amide, which is composed of a 1,2-oxazadecaline moiety and a coumarin derivative. Compound **72** exhibited antibacterial activity against *Mycobacterium tuberculosis* H37Ra with an MIC of 12.5 µg/mL [28]. A new epidithiodiketopiperazine, pretrichodermamide G (**73**), was afforded by *Trichoderma harzianum* associated with the medicinal plant *Zingiber officinale* [29]. Although compound **73** is quite similar to compound **72**, no chemical transformation under alkaline conditions was reported.

The investigation of *Aspergillus tamarii* FR02 led to the isolation of a new cyclic pentapeptide, disulfide cyclo-(Leu-Val-Ile-Cys-Cys), named malformin E (**74**). *A. tamarii* FR02 was isolated from the root of *Ficus carica*. Malformin E (**74**) exhibited cytotoxic activities against MCF-7, A549 and HepG2 with IC_50_ values of 0.65, 2.42 and 36.02 μM, respectively. Malformin E (**74**) also showed antimicrobial and antifungal activities against *Bacillus subtilis*, *Staphylococcus aureus*, *Pseudomonas aeruginosa*, *Escherichia coli*, *Penicillium chrysogenum*, *Candida albicans* and *Fusarium solani* with MIC values ranging from 0.45 to 7.24 μM [30].

Six pentacyclic diketopiperazines, brocazines A-F (**75–80**), were discovered from *Penicillium brocae* MA-231, a fungus obtained from the fresh tissue of the marine mangrove plant *Avicennia marina*. Brocazines A (**75**), B (**76**), E (**79**) and F (**80**) were cytotoxic to a panel of nine tumor cell lines with IC_50_ values ranging from 0.89 to 9.0 μM. [31]. A culture of *Phoma* sp. OUCMDZ-1847 afforded one new phomazine C (**81**), which should be biogenetically generated from the same precursor as compounds **41** and **42** [19]. *Penicillium janthinellum* HDN13-309 produced epipolythiodioxopiperazines, penicisulfuranols A−C (**82**–**84**), together with compounds **45**–**47**. Compounds **82**–**84** exhibited cytotoxicity against HeLa and HL-60 with IC_50_ of 0.1–3.9 μM [21].

Brocazine G (**85**), a new diketopiperazine, along with compounds **43** and **44** was obtained from *Penicillium brocae* MA-231 associated with the fresh tissue of the marine mangrove plant *Avicennia marina*. It showed cytotoxicity against A2780 with an IC_50_ of 59 μM. Brocazine G (**85**) also showed inhibitory activity against *E. coli*, *Aeromonas hydrophilia* and *V. harveyi* with the same MIC of 32.0 μg/mL [20]. Five new epipolysulfanyldioxopiperazines, gliocladines A–E (**86**–**90**), were isolated from *Gliocladium roseum* 1A, a fungal strain isolated from submerged wood collected from fresh water in Yunnan Province, P. R. China. Both compounds **86** and **87** are dimers with each monomer being derived from l-alanine and l-tryptophan, while each of compounds **88**–**90** is a diketopiperazine with an indole ring connected to the 1,2,3,3a,8,8a-hexahydropyrrolo[2,3-*b*]indole. These compounds exhibited nematicidal activities toward *C. elegans*, *P. redivivus* and *B. xylophilus* with ED_50_ values ranging from 25 to 250 μg/mL [32].

An analog of compounds **86** and **87**, 6-Formamide-chetomin (**91**), was obtained from a culture of *Chaetomium* sp. M336, isolated from the plant *H. serrata* (Thunb. ex Murray) Trev. Compound **91** was cytotoxic to HeLa, SGC-7901 and A549 cells with IC_50_ values of 21.6–27.1 μM. It exhibited activity against *Escherichia coli*, *Staphylococcus aureus*, *Salmonella typhimurium* ATCC 6539 and *Enterococcus faecalis* with the same MIC of 0.78 μg/mL [33].

### 2.3. Sulfoxide (R-SO-R′) and Sulfone (R-SO_2_-R′)

An indole alkaloid with a rare methylsulfonyl unit, 21-*Epi*-taichunamide D (**92**), was obtained from *Aspergillus versicolor* F210 (*Lycoris radiata*). The strain was isolated from the bulbs of *Lycoris radiata* collected from Yichang City in Hubei Province, P. R. China. Compound **92** inhibited anticancer activity toward HL-60 and A549 cells with IC_50_ values of 26.8 and 32.5 μM, respectively [34].

## 3. Polyketides

### 3.1. Sulfide

**Figure 4:**
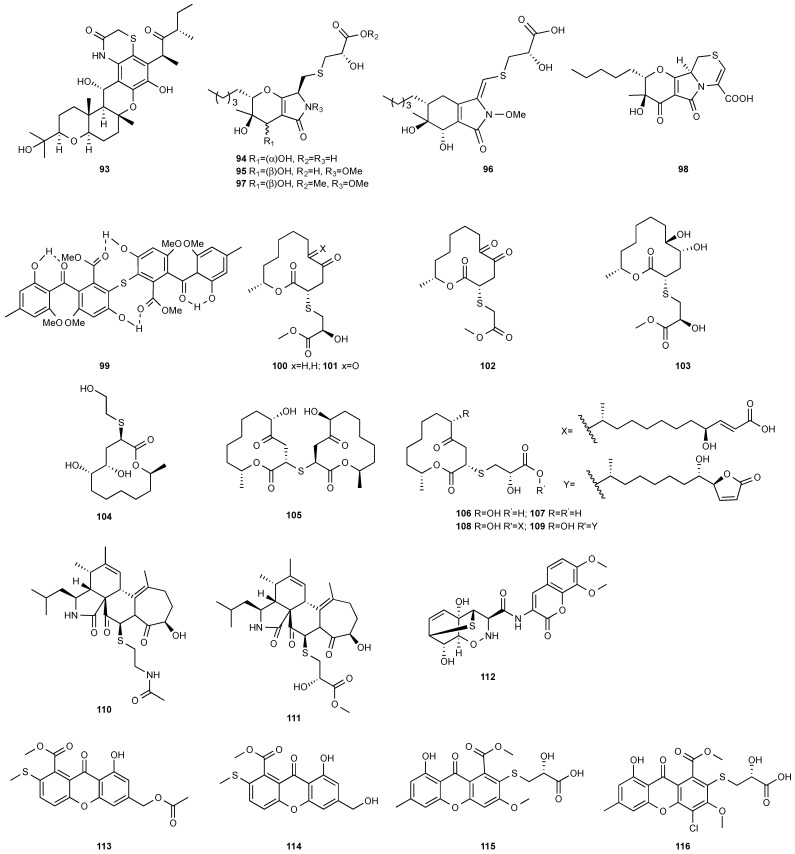
Structures of compounds **93**–**116**.

A new cytotoxic compound, isocochlioquinones D (**93**) (Figure 4), was purified from *Bipolaris sorokiniana* A606. The endophytic fungus was isolated from the medicinal plant *Pogostemon cablin*, also known as patchouli or “Guanghuoxiang” in traditional Chinese medicine (TCM) [35]. Isocochlioquinones D (**93**) is a hybrid of a polyketide and a sesquiterpenoid with a rare benzothiazin-3-one moiety. Compound **93** demonstrated antiproliferative activity toward SF-268, MCF-7, NCI-H460 and HepG-2 with IC_50_ values of 32.8, 28.3, 42.6 and 38.6 µM, respectively.

*Paraphaeosphaeria neglecta* FT462 yielded paraphaeosphaerides E (**94**), F (**95**), H (**96**) and methyl ester of paraphaeosphaeride F (**97**) [36]. *P. neglecta* FT462 was isolated from the Hawaiian plant *Lycopodiella cernua*, synonym *Palhinhaea cernua* (Lycopodiaceae). Paraphaeosphaeride E (**94**) was active against *E. coli* JW2496 at 20 μg/mL. Paraphaeosphaeride E (**94**) inhibited nuclear factor kappa B (NF-κB) with an IC_50_ of 7.1 μM. Paraphaeosphaerides E (**94**) and F (**95**) also showed inducible nitric oxide synthase (iNOS) with IC_50_ values of 47.9 and 43.2 μM, respectively. Paraphaeosphaeride A (**98**) with the unique 4-pyranone-γ-lactam-1,4-thiazine moiety was obtained from *P. neglecta* FT462 [37].

The first natural sulfur-containing benzophenone dimer, named guignasulfide (**99**), was isolated from the culture of *Guignardia* sp. IFB-E028, an endophytic fungus residing in the healthy leaves of *Hopea hainanensis.* Guignasulfide (**99**) exhibited cytotoxicity against HepG2 with an IC_50_ of 5.27 μM. It also showed antimicrobial activity against *Helicobacter pylori* with an MIC of 42.9 μM [38].

*Cladosporium cladosporioides* MA-299 yielded four 12-membered macrolides, thiocladospolides A-D (**100**–**103**). *C. cladosporioides* MA-299 is an endophytic fungus obtained from the leaves of the mangrove plant *Bruguiera gymnorrhiza*. Thiocladospolide A (**100**) was active against *E. tarda*, *E. ictarda* and *C. glecosporioides* with MIC values of 1, 8 and 2 μg/mL, respectively. Thiocladospolide B (**101**) was active against *C. glecosporioides*, *P. piricola Nose* and *F. oxysporum f. sp.cucumerinum* with MIC values of 2, 32 and 1 μg/mL, respectively. Thiocladospolide C (**102**) was active against the same three strains as **101** with MIC values of 1, 32 and 32 μg/mL, respectively. Thiocladospolide D (**103**) was active against *E. ictarda*, *C. glecosporioides*, *P. piricola* Nose and *F. oxysporum f. sp.cucumerinum* with MIC values of 1, 1, 32, and 1 μg/mL, respectively [39]. The investigation of the mangrove-derived fungus *Cladosporium* sp. SCNU-F0001 afforded a new 12-membered macrolide, thiocladospolide E (**104**) [40]. A mangrove-derived fungus, *Cladosporium oxysporum*, yielded five 12-membered macrolides, thiocladospolides F–J (**105**–**109**), and they showed a broad spectrum of antimicrobial activity with MIC values ranging from 4 to 32 μg/mL [41].

Two cytochalasan analogs, cyschalasins A (**110**) and B (**111**), were obtained from *Aspergillus micronesiensis*, which was isolated from the root of the traditional Chinese medicinal plant *Phyllanthus glaucus* collected from LuShan Mountain, Jiangxi Province, P. R. China. Cyschalasins A (**110**) and B (**111**) exhibited cytotoxicity against HL60, A549, Hep3B, MCF-7 and SW480 with IC_50_ values in the range of 3.0 to 19.9 μM except for **110**, which was inactive toward A549 at 20 μM. Cyschalasins A (**110**) and B (**111**) also demonstrated antimicrobial activity with MIC_50_ values ranging from 10.6 to 94.7 μg/mL [42].

An amide of a coumarin moiety and l-phenylalanine-derived 1,2-oxazadecaline moiety, trichodermamide G (**112**), was isolated from *Trichoderma harzianum* D13. The fungal strain was isolated from the internal tissues of the root of *Excoecaria agallocha*, distributed in the mangrove regions of various parts of India [43].

Two sulfur-containing xanthones, sydoxanthone A (**113**) and sydoxanthone B (**114**), were purified from *A. sydowii*, occurring in the liverwort *Scapania ciliata* S. Lac. Sydoxanthone B (**114**) was active on the concanavalin A-induced and lipopolysaccharide (LPS)-induced proliferation of mouse splenic lymphocytes with IC_50_ of 22.53 and 15.30 μg/mL, respectively [44]. Sydoxanthones D (**115**) and E (**116**) were discovered from *Pseudopestalotiopsis theae*, which was isolated from the leaves of *Caloncoba welwitschii* [45].

### 3.2. Disulfide

**Figure 5:**
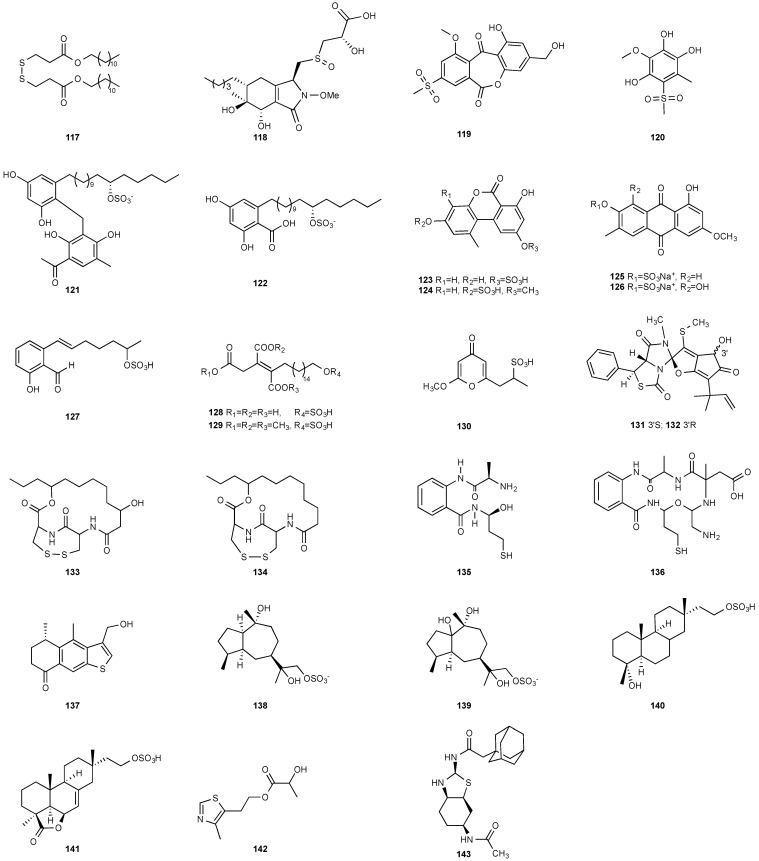
Structures of compounds **117**–**143**.

A new natural compound, a symmetrical disulfide dimer dodecyl 3,3″-dithiodipropionate (**117**) (Figure 5), was isolated from the ethyl acetate extract of fermentation broth of an endophytic fungus, *Sphaceloma* sp. LN-15. The fungal strain was isolated from the leaves of *Melia azedarach* L., commonly known as the chinaberry tree, pride of India, Persian lilac, and some other names [46]. The structure of **117** was determined by NMR and MS and was further confirmed by chemical synthesis.

### 3.3. Sulfoxide

LC-UV/MS-based metabolomics analysis of the Hawaiian endophytic fungus *Paraphaeosphaeria neglecta* FT462 led to the identification of unique mercaptolactated γ-pyranol–γ-lactams, paraphaeosphaerides G (**118**). The fungal strain was isolated on potato dextrose agar (PDA) medium from a healthy leaf of the Hawaiian indigenous plant *Lycopodiella cernua* (L.) Pic. Serm, which was collected in the Mokuleia Forest Reserve in 2014 [36].

### 3.4. Sulfones

Two new polyketides modified with a rare methyl sulfonyl group, neosartoryone A (**119**) and 3-methoxy-6-methyl-5-(methylsulfonyl)benzene-1,2,4-triol (**120**), were isolated from *Neosartorya udagawae* HDN13-313 cultivated with the DNA methyltransferase inhibitor 5-azacytidine. *N. udagawae* HDN13-313 was isolated from the root of the mangrove plant *Aricennia marina* [47]. Compound **119** decreased the lipid accumulation elicited by oleic acid at 10 μM.

### 3.5. Sulfates and Sulfonates

Two new alkyl sulfate-containing aromatic compounds, penixylarins B (**121**) and D (**122**), were isolated from a mixed culture of the Antarctic deep-sea-derived fungus *Penicillium crustosum* PRB-2 and the fungus *Xylaria* sp. HDN13-249 [48]. *Xylaria* sp. HDN13-249 was isolated from the root of *Sonneratia caseolaris* collected from the mangrove conservation area of Hainan, P. R. China. Penixylarins B (**121**) showed weak antibacterial activity against *Bacillus subtilis* with an MIC_50_ of 100 μM.

Alternariol 5-*O*-sulfate (**123**) and alternariol 5-*O*-methyl ether-4′-*O*-sulfate (**124**) were produced by *Alternaria* sp., which was isolated from fresh healthy leaves of the wild Egyptian medicinal plant *Polygonum senegalense Meisn*. (Polygonaceae) [49]. Alternariol 5-*O*-sulfate (**123**) was cytotoxic against L5178Y with an EC_50_ of 4.5 μg/mL. Compound **123** also showed inhibition toward a panel of protein kinases at the micromolar level.

The extracts of cultures grown in liquid or on solid rice media of the fungal endophyte *Ampelomyces* sp. isolated from the medicinal plant *Urospermum picroides* exhibited considerable cytotoxic activity against L5178Y cells. The extract obtained from liquid cultures afforded two sulfated anthraquinones, macrosporin-7-*O*-sulfate (**125**) and 3-*O*-methylalaternin-7-*O*-sulfate (**126**) [50]. However, neither compound showed any cytotoxic or antimicrobial activities.

A 2-hydroxyl 6-alkylated benzaldehyde derivative, pestalols E (**127**), was isolated from the endophytic fungus *Pestalotiopsis* sp. AcBC2, which was derived from the Chinese mangrove plant *Aegiceras corniculatum*, commonly known as black mangrove or river mangrove [51].

Oreganic acid (**128**) and its trimethyl esters (**129**) were obtained from the extract of an endophytic fungus MF6046 isolated from living leaves of *Berberis oregano* [49]. Oreganic acid (**128**) is a highly potent and specific farnesyl protein transferase (FPTase) inhibitor (IC_50_ = 14 nM) [49].

A novel metabolite containing a sulfonate group, fusaodavinvin (**130**), was isolated from an endophytic fungus *Fusarium* sp. (CTGU-ZL-34). The fungal strain was isolated from a healthy plant *Davidia involucrata*. Compound **130** displayed inhibitory activity against A549, HepG2, Caski and MCF-7 cell lines with IC_50_ values of 11.5, 15.3, 15.2 and 60.5 μg/mL, respectively [53].

## 4. Hybrids

### 4.1. Sulfides

A fungal strain *Pestalotiopsis* sp. HS30 was isolated from the fresh stems of *Isodon xerophilus* collected at Kunming Botanical Garden, Yunnan Province, P. R. China [54]. Pestaloamides A (**131**) and B (**132**), two novel alkaloids featuring an unprecedented spiro[imidazothiazoledione-alkylidenecyclopentenone] scaffold, were obtained from the cultures of *Pestalotiopsis* sp. HS30. Compounds **131** and **132** were derived from a polyketide and a Phe-Cys dipeptide together with C_2_ and C_5_ moieties. Both compounds could enhance the cell surface engagement of NKG2D ligands in HCT116 cells at 40 μM [54].

### 4.2. Disulfides

PM181110 (**133**) was a new depsipeptide obtained from *Phomopsis glabrae*, which was isolated from the leaves of *Pongamia pinnata* (Fabaceae) [55]. Compound **133** was derived from two molecules of l-cysteine and one C_12_ polyketide. It exhibited potent cytotoxic activity toward 40 human cancer cell lines at the nanomolar level (mean IC_50_ = 89 nM) and 24 human tumor xenografts with the mean IC_50_ of 245 nM [55].

FE399 (**134**), a dehydroxylated **133**, was isolated from *Ascochyta* sp. AJ 117309, an endophytic strain separated from a raw leaf of *Taxus cuspidata var. nana* Rehd. [56]. Compound **134** also demonstrated potent cytotoxic activity against SWS948, K562T, Colon26, CHO-K1 and P388 cells with IC_50_ values ranging from 75 to 400 ng/mL [56].

### 4.3. Thiols

*Fusarium chlamydosporium*, an endophytic fungus isolated from the leaves of *Anvillea garcinii* (Burm.f.) DC. (Asteraceae), produced a new benzamide derivative, fusarithioamide A (**135**), which is composed of a 2-aminobenzamide moiety, an l-alanine and a 3-mercaptopropan-1-ol moiety derived from l-cysteine. Compound **135** displayed cytotoxicity against SK-MEL, KB, BT-549 and SKOV-3 cells with IC_50_ values of 9.3, 7.7, 0.4 and 0.8 μM, respectively. It was also active against *S. aureus*, *B. cereus*, *E. coli*, *P. aeruginosa* and *C. albicans* with MIC values of 4.4, 3.1, 6.9, 100 and 2.6 μg/mL, respectively [57].

Fusarithioamide B (**136**), a new aminobenzamide derivative with an unprecedented carbon skeleton, was separated from an EtOAc extract of *Fusarium chlamydosporium* isolated from *Anvillea garcinii* (Burm.f.) DC. leaves (Asteraceae) [58]. Fusarithioamide B (**136**) displayed antifungal activity toward *C. albicans* with an MIC of 1.9 μg/mL. It also showed high antibacterial activity against *E. coli*, *S. aureus* and *B. cereus* with MIC values of 3.4, 2.9 and 3.9 μg/mL, respectively. Compound **136** exhibited cytotoxic activity toward BT-549, MCF-7, HCT-116, SKOV-36, KB and SK-MEL with IC_50_ values of 0.09, 0.21, 0.59, 1.23, 6.9 and 11.2 µM, respectively [58].

## 5. Terpenoids

### 5.1. Sulfide/Thiophene

Leptosphin A (**137**), a new sesquiterpenoid with a benzo[b]thiophene moiety, was obtained from a culture of Leptosphaeria sp. XL026 isolated from the leaves of *Panax notoginseng* [59]. Leptosphin A (**137**) displayed antifungal and antibacterial activity with MIC values ranging from 25 to 100 μg/mL [59].

### 5.2. Sulfates

An endophytic fungus S49 was isolated from the bark of *Cephalotaxus hainanensis*, known as Hainan plum-yew. S49 afforded two new sesquiterpenoids 1,10,11,12-guaianetetrol (**138**) and 1,10,11,12-guaianetetrol (**139**) [60]. Two new isopimarane diterpenoids, 16-*O*-sulfo-18-norisopimar-7-en-4α,16-diol (**140**) and 9-deoxy-hymatoxin A (**141**), were isolated from the culture broth of an endophytic fungus, *Xylaria* sp. YM 311647, obtained from *Azadirachta indica*. Compounds (**140**) and (**141**) were active against *C. albicans* YM 2005, *A. niger* YM 3029, *P. oryzae* YM 3051, *F. avenaceum* YM 3065 and *H. compactum* YM 3077 with MIC values in the range of 32–128 μg/mL, while compound **141** had the same MIC of 16 μg/mL toward *C. albicans* and *P. oryzae* [61].

## 6. Others

A new thiazole derivative, colletotricole A (**142**), was obtained from *Colletotrichum gloeosporioides* A12, an endophytic fungus derived from *Aquilaria sinensis* [62]. A sulfur-containing anticandidal compound, *N*-[(2*S*,3a*R*,6*S*,7a*S*)-6-acetamido-octahydro-l,3-benzothiazoi-2-yl]2-(adamantan-l-yl) acetamide (**143**), was isolated from *Emericella* sp. from *Azadirachta indica* [63].

## 7. Discussion and Conclusions

**Figure 6:**
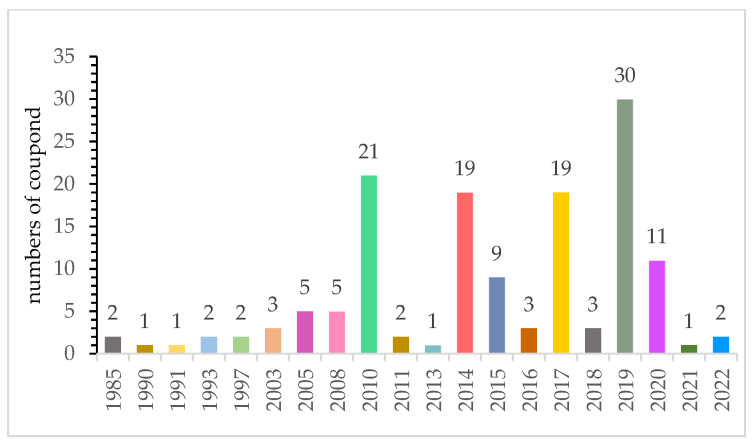
Annual numbers of sulfur-containing compounds identified from 1985 to 2022. (Keywords: sulfur-containing compound, plant endophytic fungi; Databases: SciFinder, PubMed).

**Figure 7:**
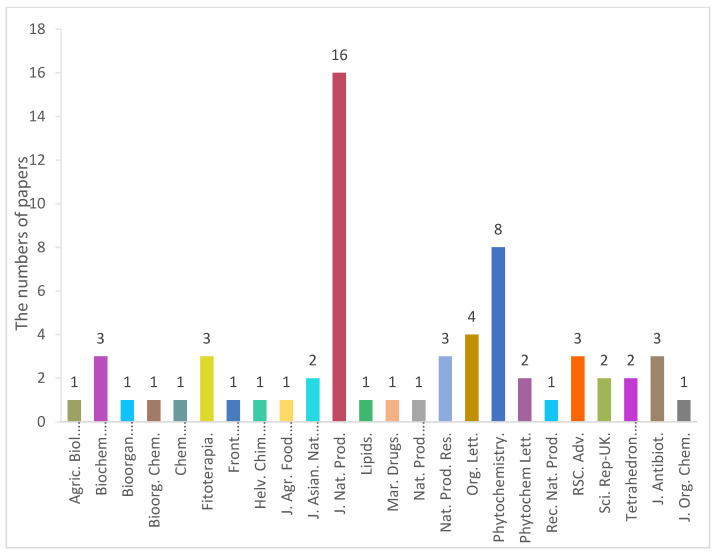
The journal names and numbers for the papers that reported sulfur-containing compounds.

From 1985 to March 2022, 143 new sulfur-containing compounds were obtained from endophytic fungi. This review summarized the fungal producers, host plants, chemical structures and biological activities of these fungal metabolites (Table 1). The majority of these compounds (109 out of 143) were reported in 2010, 2014, 2015, 2017, 2019 and 2020 (Figure 6). There was a trend that more sulfur-containing compounds were reported in recent years except 2021. Only one sulfur-containing compound was reported in 2021, most likely due to the outbreak of COVID-19 in 2020. A total of 24 journals reported these compounds (Figure 7). The *J. Nat. Prod.* has published the highest number of papers (16) that reported sulfur-containing compounds, followed by *Phytochemistry* (8) (Figure 7). This is not unexpected because both *J. Nat. Prod.* and *Phytochemistry* are prominent natural product journals.

**Figure 8:**
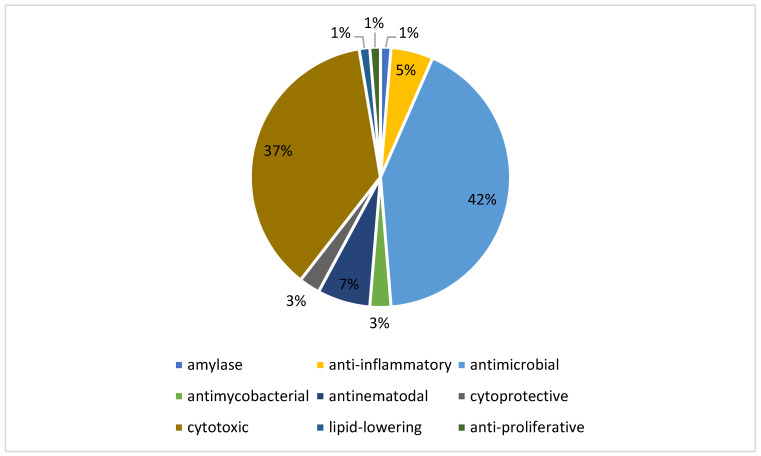
The percentages of the biological activity among sulfur-containing compounds from endophytic fungi.

**Figure 9:**
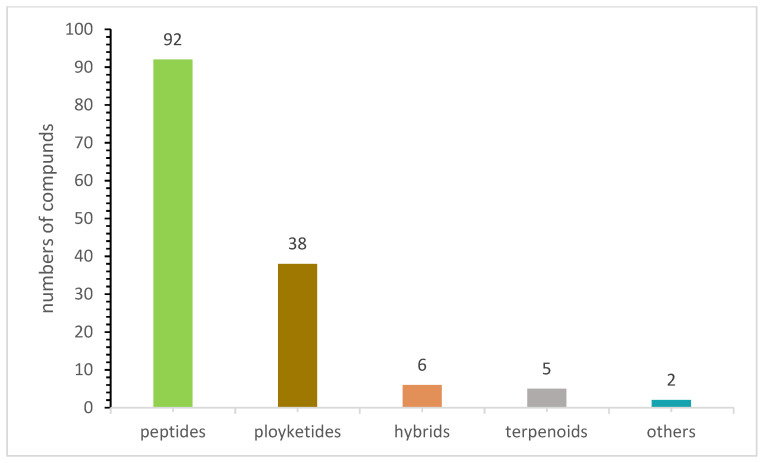
The structural classes of sulfur-containing compounds isolated from endophytic fungi.

These sulfur-containing compounds demonstrate functional and structural diversity and exhibited many bioactivities. Among the reported biological activities, 42% of these compounds were antimicrobial, while 37% were cytotoxic (Figure 8), which is not surprising because the majority of the FDA-approved antimicrobial and anticancer drugs are either natural products or derived from natural products. For example, Secoemestrin D (**69**), a diketopiperazine, was very active against a panel of seven cancer cell lines with IC_50_ values ranging from 0.06 to 0.24 μM [27], while PM181110 (**133**) [55] and FE399 (**134**) [56], hybrids of polyketides and peptides, exhibited potent anticancer activity with IC_50_ values at the nM level. These compounds also possess other bioactivities. For instance, oreganic acid (**128**), a fatty acid derivative, inhibited FPTase with an IC_50_ of 14 nM [49]. The majority of sulfur-containing compounds (92) were peptides, followed by polyketides (38), hybrids (6), terpenoids (5) and others (2) (Figure 9). All 92 of these peptides are diketopiperazines, and the sulfur atoms in these molecules are mainly derived from l-cysteine that contains a reactive sulph-hydryl group.

## 8. Prospects

Some plants are rich in sulfur, for example, allium vegetables, legumes and cruciferous plants. These plants should be great sources of endophytic fungi that produce sulfur-containing compounds. Large amounts of sulfur are released during volcanic eruptions. Hence, plants in volcanic areas and hot springs might also be excellent sources for endophytic fungi producing sulfur-containing compounds.

Most of the compounds reviewed in this article were tested for their antimicrobial and antiproliferative or anticancer activities. We believe that other biological properties could be identified if fungal metabolites were evaluated in a broader range of biological settings. For example, sinuxylamides A and B were obtained from *Xylaria* sp. FM1005, an endophytic fungus isolated from *Sinularia densa* (leather coral) collected in the offshore region of the Big Island, Hawaii [64]. Sinuxylamides A and B showed no antibacterial activity or cytotoxicity at 40 μM, but they strongly inhibited the binding of fibrinogen to purified integrin IIIb/IIa in a dose-dependent manner with IC_50_ values of 0.89 and 0.61 μM, respectively.

Diketopiperazines are expected to be biosynthetically assembled from two amino acid building blocks by nonribosomal peptide synthetases [65]. On the other hand, the biogenesis of many sulfur-containing compounds remains incompletely understood. For example, the structures of compounds **20** [10], **40** [18], **98** [37], **136** [58], **142** [62] and **143** [63] are unique. It would be interesting to investigate how these molecules are biogenetically synthesized. Presumably, the 4,5-dihydrooxepine ring in **20** is derived from the benzene ring of l-phenylalanine through ring expansion. On the other hand, the spiro[cyclopenta[*b*]pyrrole-5,2′-furan] moiety in **40** might be formed through the constriction of the benzene ring of l-phenylalanine followed by the merge of the octahydrocyclopenta[*b*]pyrrole ring with an isoprenyl (C_5_) group. We previously isolated compound **98** [37]. The precursor of the side chain at the 14-position in compound **98** could be l-cysteine, which is converted to mercaptolactate. The nucleophilic addition of the mercaptolactate thiol to C-14 of paraphaeosphaeride C generates an intermediate that is oxidized to another intermediate. It is also plausible that the second intermediate is generated from mercaptopyruvate and paraphaeosphaeride C. The nitrogen atom in the second intermediate undergoes intramolecular nucleophilic addition to the ketone of the mercaptopyruvate moiety, leading to the formation of the third intermediate. The dehydration of the third intermediate yields the final product **98** [37]. However, the experimental details of the biosynthesis of compound **98** are still not available. Compound **136** is composed of five fragments, including a 2-amino benzoic acid moiety, an l-alanine, a 2-amino-2-methylsuccinic acid fragment that might be derived from an isoprenyl group (C_5_), and l-glycine and l-cysteine-derived 3-mercaptopropanoic acid moieties. Compound **142** carries a 2-hydroxyl propanoic acid ester. The thiazole ring in **142** is probably derived from acetate and l-cysteine, while the linker (-CH_2_-CH_2_-) might be derived from another acetate. It would be interesting to investigate how **142** is synthesized biogenetically. Investigating the biosynthesis of diamond-like compound **143** should be very challenging and interesting. Recent advances in genome mining and synthetic biology offer new opportunities to discover new natural products [66]. It becomes routine to sequence the (meta)genomes of fungal isolates, and capable bioinformatics tools (e.g., antiSMASH fungal version) [67] are increasingly available for identifying potential biosynthetic gene clusters (BGCs) of fungal natural products [68]. These predicted BGCs can suggest new chemotypes, enzymology and bioactivities. Subsequently, native and engineered BGCs can be expressed in multiple synthetic biology chasses, such as *Aspergillus nidulans* [69] and Saccharomyces cerevisiae [70]. In this regard, biosynthetic research is critical for laying the basis for the genome mining of BGCs of new fungal sulfur-containing compounds with bioactivities, particularly those whose biogenesis remains unclear.

## Figures and Tables

**Table 1:** Sulfur-containing compounds isolated from plant endophyte fungi.

Compound Structures	Producing Strain	Host Plant etc.	Bioactivity	Reference(s)
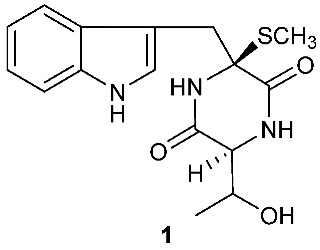	*Bionectria* sp. Y1085	*Huperzia serrata*	Antibacterial	[4]
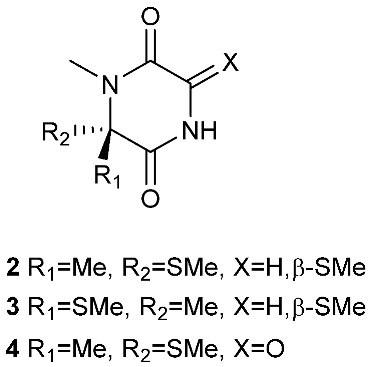 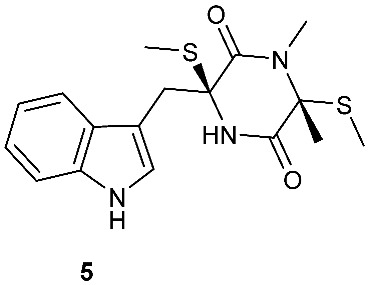 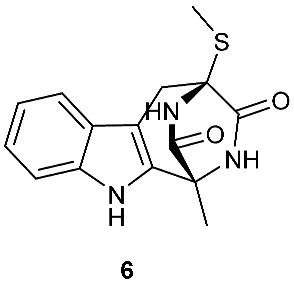	*Lasiodiplodia pseudotheobromae*	Flower of *Illigera rhodantha* (Hernandiaceae)	Antibacterial (**5**)	[5]
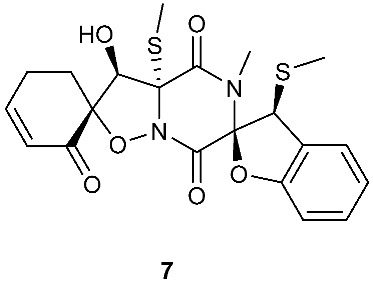 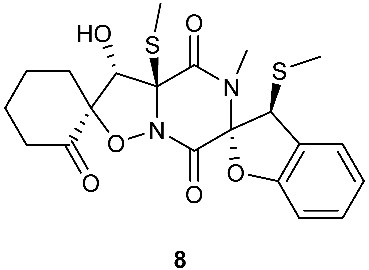	*Botryosphaeria mamani*	Fresh leaves of *Bixa orellana* L. (Bixaceae)	Anticancer	[6]
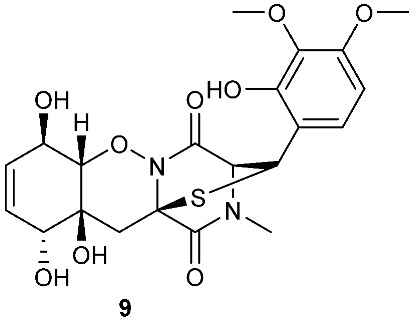	*Penicillium raciborskii* (TRT59)	*Rhododendron tomentosum*		[7]
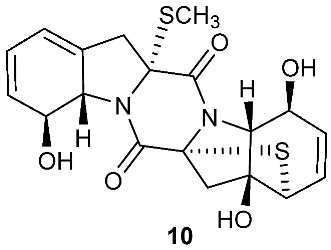 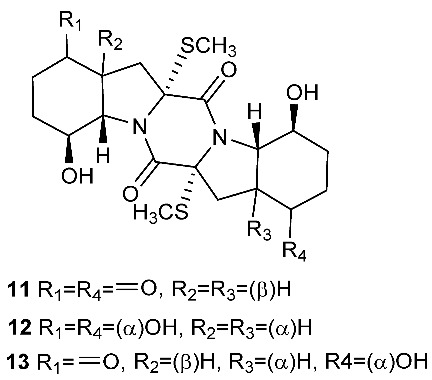 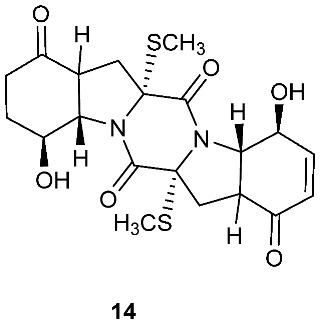 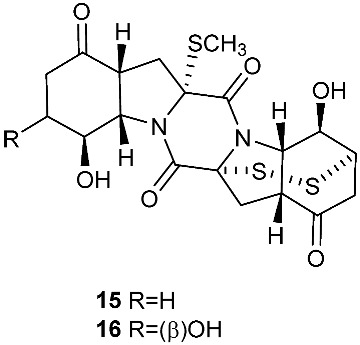 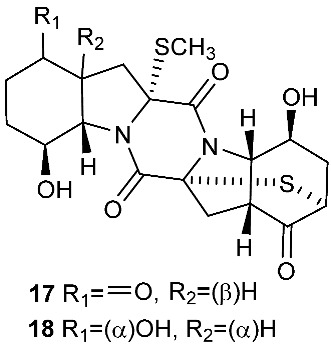	*Epicoccum nigrum*	Leaves of *Lysidice rhodostegia*	Inhibition of β- Glucuronidase release (**11** and **15**)	[8]
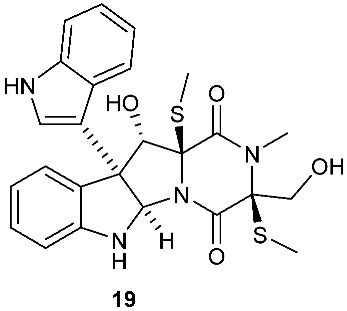	*Tilachlidium* sp. (CANU-T988)	Decaying wood sample collected in Christchurch	Cytotoxicity	[9]
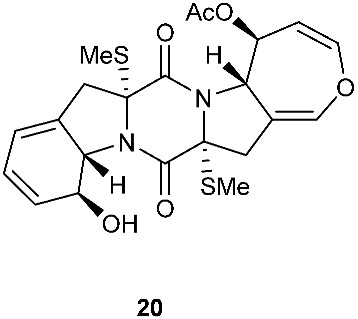	*Aspergillus terreus* BCC 4651	Tree hole	Weak antimycobacterial activity	[10]
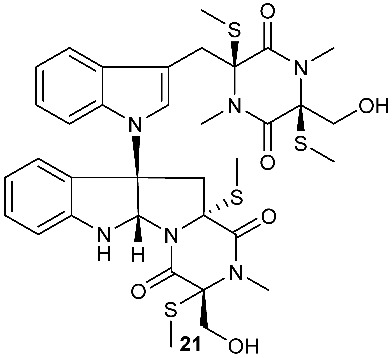 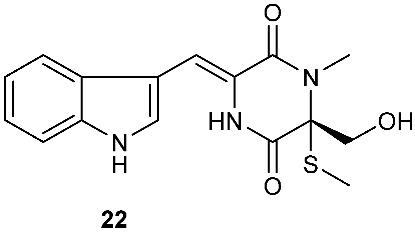 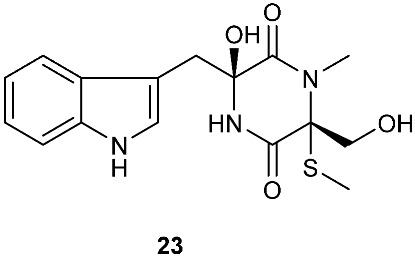	*Chaetomium* sp. 88194	*Cymbidium goeringii*	Cytotoxicity (**21**)	[11]
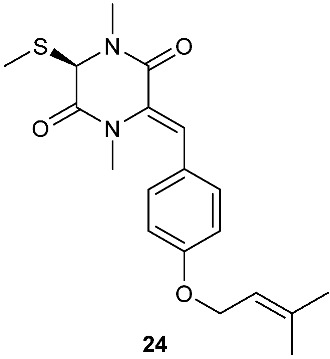 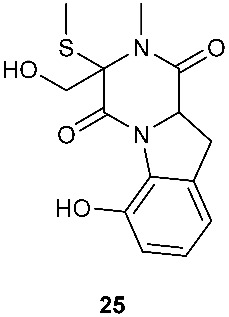	*Penicillium crustosum* and *Colletotrichum gloeosporioides*, respectively	*Viguiera robusta*		[12]
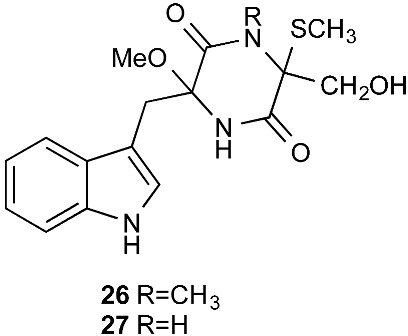	*Chaetomium* sp. SYP-F7950	*Panax notoginseng*	Cytotoxic (**26**)	[13]
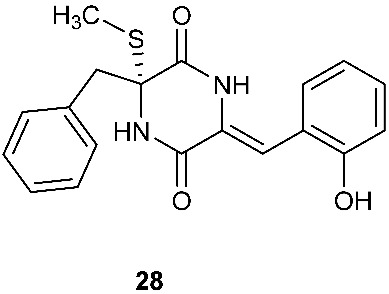 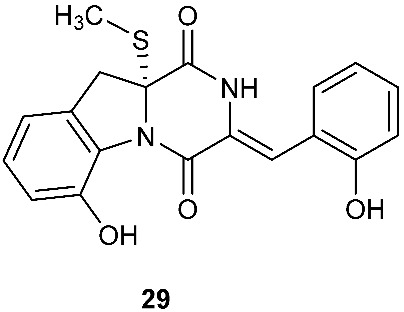 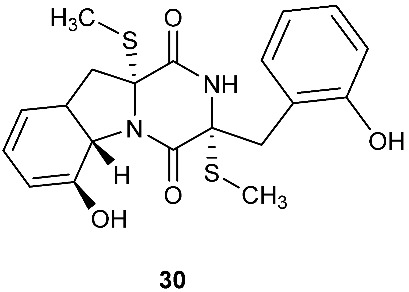 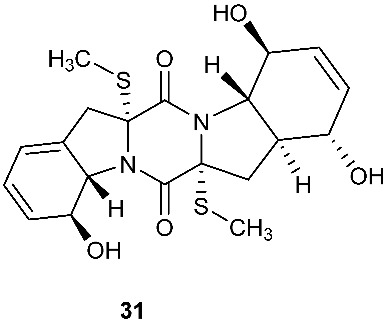	*Penicillium brocae* MA-231	Fresh tissue of the marine mangrove plant *Avicennia marina*	Antibacterial (**30** and **31**)	[14]
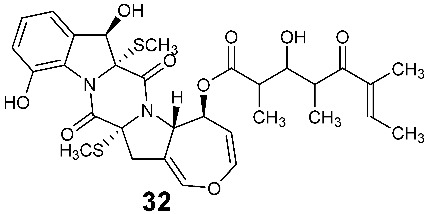 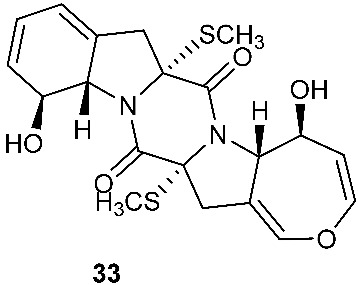	*Menisporopsis theobromae* BCC 3975	Seed	Antimycobacterial Cytotoxic (**32**)	[15]
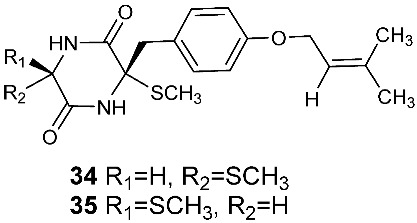	*TolypocJadium* sp.	*Quercus virginiana Miller*	PAF inhibition (**35**)	[16]
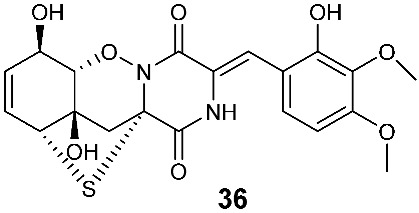 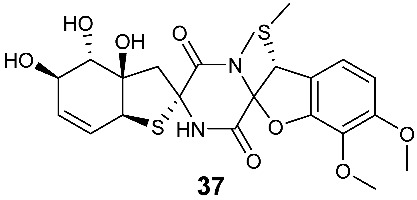 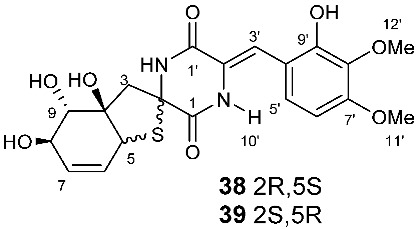	*Penicillium janthinellum* HDN13-309	Root of *Sonneratia caseolaris*	Cytoprotective (**38** and **39**)	[17]
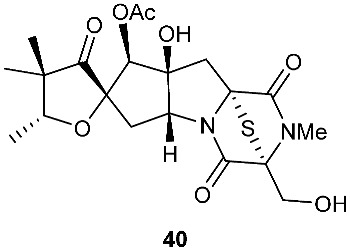	*Phoma lingam* isolate Leroy	Rapeseed		[18]
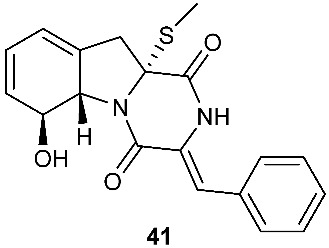 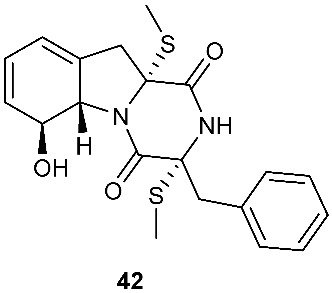	*Phoma* sp. OUCMDZ-1847	Mangrove plant *Kandelia candel*	Cytotoxic (**42**)	[19]
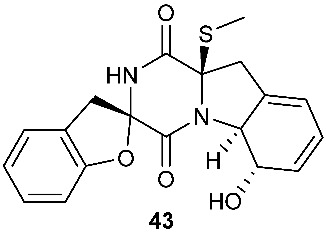 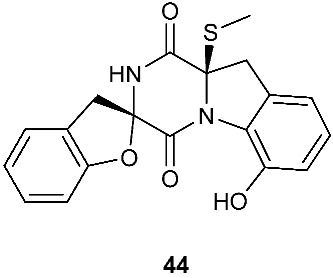	*Penicillium brocae* MA-231	*Avicennia marina*	Antimicrobial (**43**)	[20]
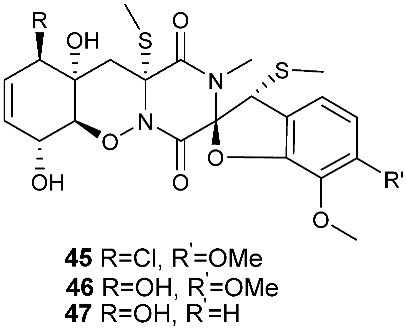	*Penicillium janthinellum* HDN13-309	Root of *Sonneratia caseolaris*		[21]
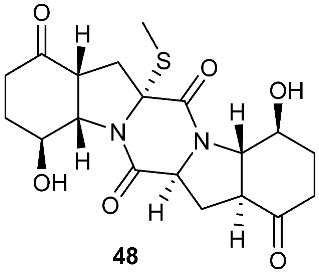 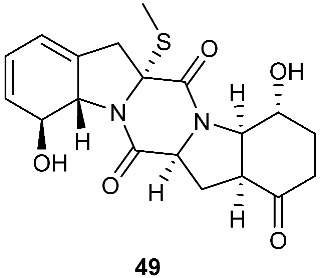 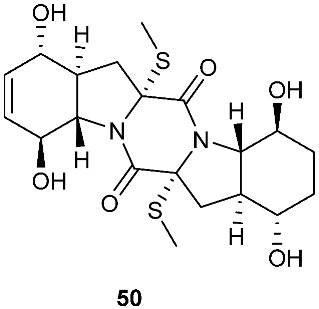 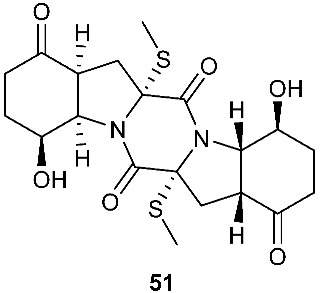 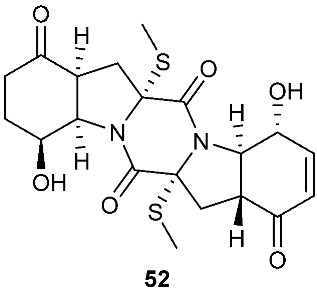	*Penicillium brocae* MA-231	Fresh tissue of the marine mangrove plant *Avicennia marina*	Antimicrobial	[22]
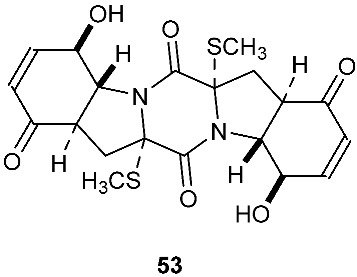 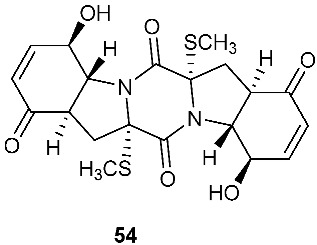	*Exserohilum holmii*	*Dactyloctenium aegyptium*		[23]
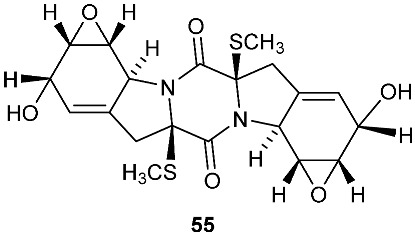	*Nigrospora sphaerica*	Germinating fescue seed		[24]
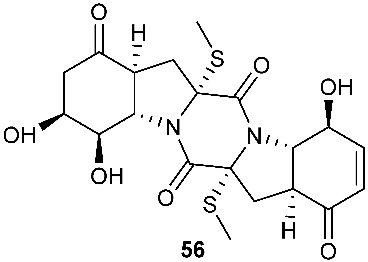 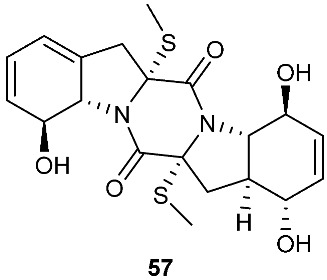 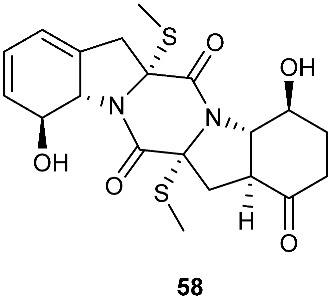	*Setosphaeria rostrata*	Fresh asymptomatic leaf tissues of the medicinal plant *Costus speciosus*	Inhibiting porcine pancreatic alpha-amylase (**57**)	[25]
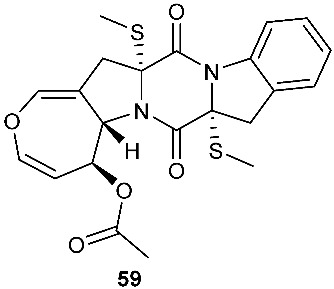	*Aspergillus versicolor* 0312	Stems of *Paris polyphylla var. yunnanensis*	Cytotoxic	[26]
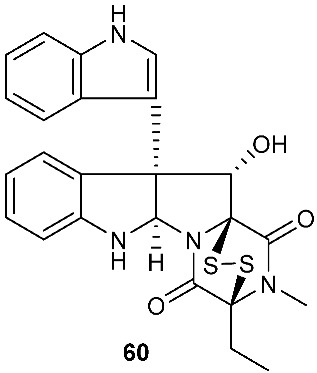	*Bionectria* sp. Y1085	*Huperzia serrata*	Antibacterial	[9]
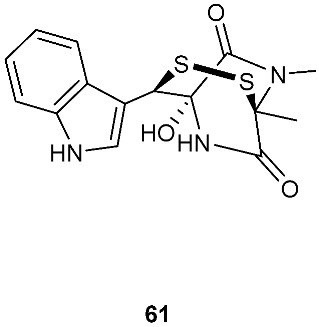	*Lasiodiplodia pseudotheobromae*	Apparently normal flower of *Illigera rhodantha* (Hernandiaceae)		[5]
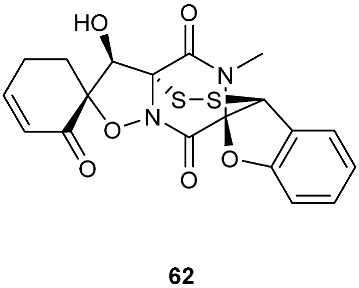	*Botryosphaeria mamani*	Fresh leaves of *Bixa orellana* L. (Bixaceae)	Cytotoxic	[6]
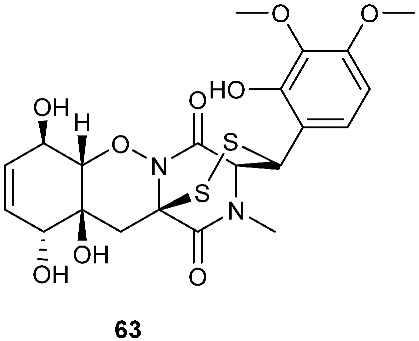 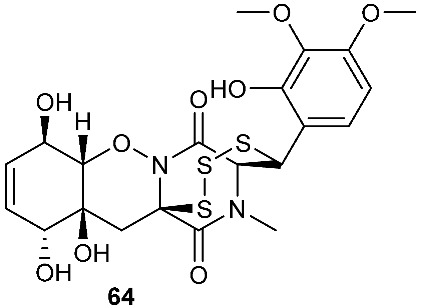	*Penicillium raciborskii* (TRT59)	*Rhododendron tomentosum*	Cytotoxic (**64**) Antifungal (**64**)	[7]
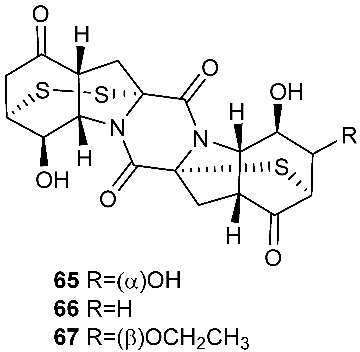 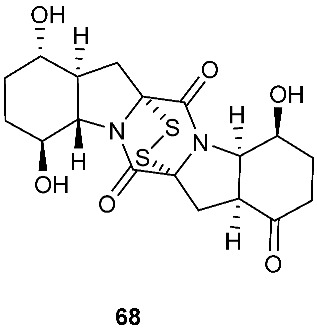	*Epicoccum nigrum*	Leaves of *Lysidice rhodostegia*	Inhibiting the release of β-glucuronidase (**67**)	[8]
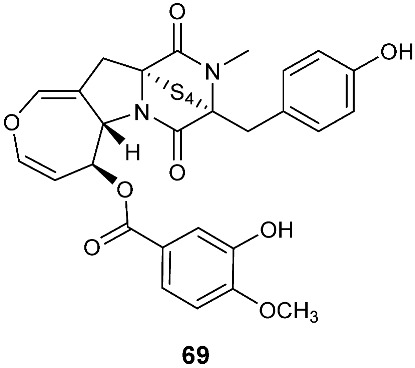	*Emericella* sp. AST0036	Healthy leaf tissue of *Astragalus lentiginosus*	Cytotoxic	[27]
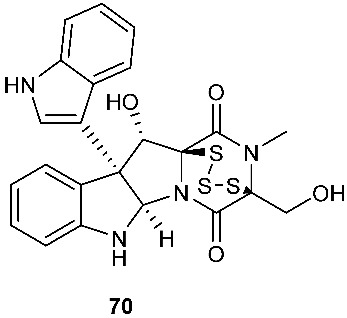 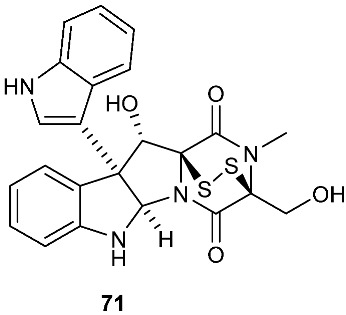	*Tilachlidium* sp. (CANU-T988)	Decaying wood sample collected in Christchurch	Cytotoxicity	[9]
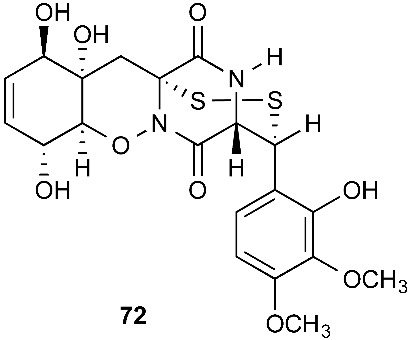	*Trichoderma* sp. BCC 5926	Bamboo leaf	Antibacterial	[28]
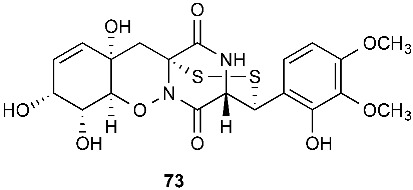	*Trichoderma harzianum*	*Zingiber officinale*		[29]
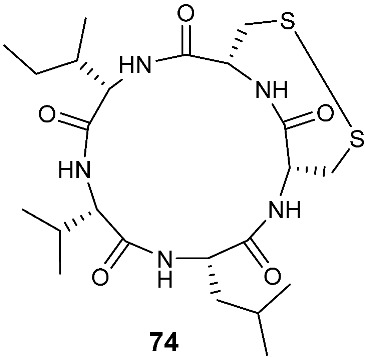	*Aspergillus tamarii*	*Ficus carica*	Cytotoxic Antimicrobial	[30]
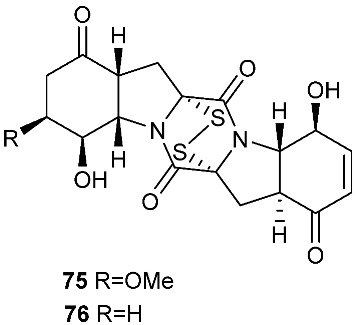 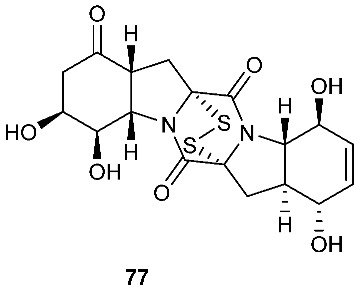 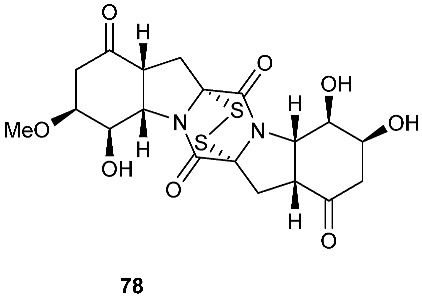 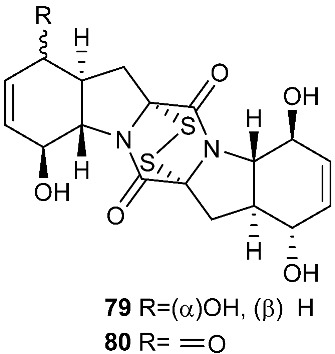	*Penicillium brocae* MA-231	Fresh tissue of the marine mangrove plant *Avicennia marina*	Cytotoxic (**75**, **76**, **79** and **80**)	[31]
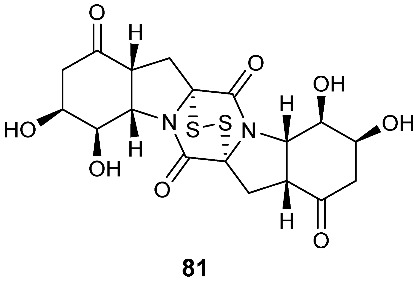	*Phoma* sp. OUCMDZ-1847	Mangrove plant *Kandelia candel*		[19]
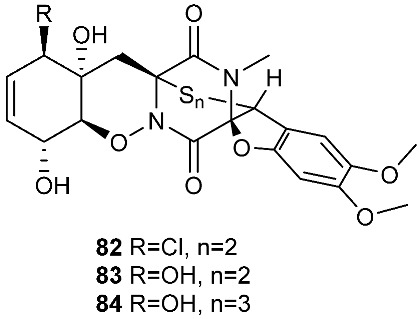	*Penicillium janthinellum* HDN13-309	Root of *Sonneratia caseolaris*	Cytotoxic	[21]
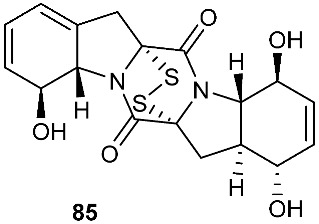	*Penicillium brocae* MA-231	Fresh tissue of the marine mangrove plant *Avicennia marina*	Cytotoxic Antimicrobial	[20]
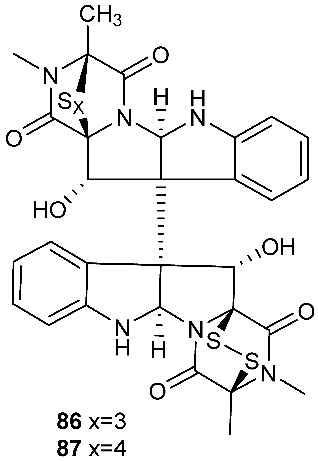 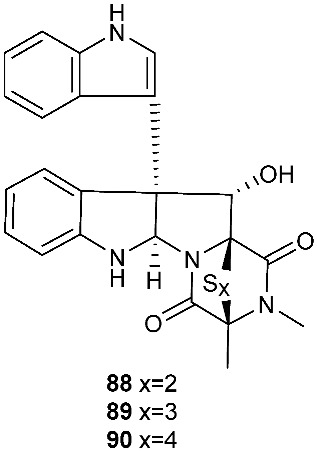	*Gliocladium roseum* 1A	Submerged wood	Nematicidal	[32]
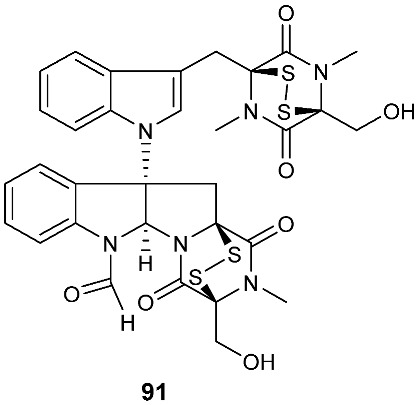	*Chaetomium* sp. M336	*Huperzia serrata Trev*	Cytotoxic Antibacterial	[33]
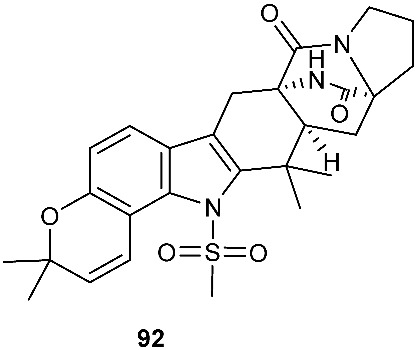	*Aspergillus versicolor* F210	Bulbs of *Lycoris radiata*	Anticancer	[34]
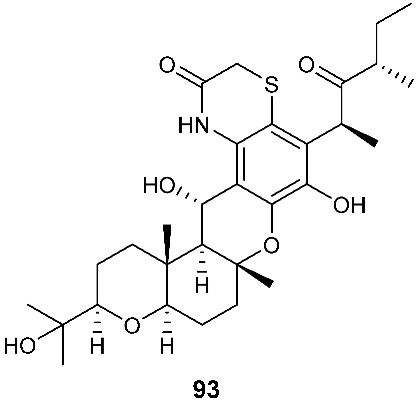	*Bipolaris sorokiniana* A606	*Pogostemon cablin*	Antiproliferative	[35]
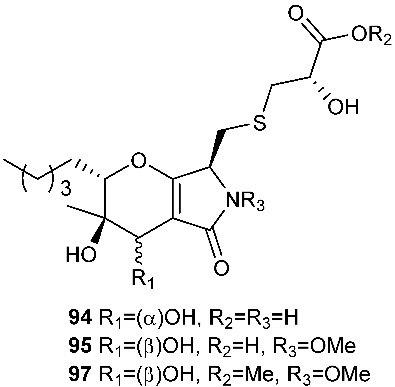 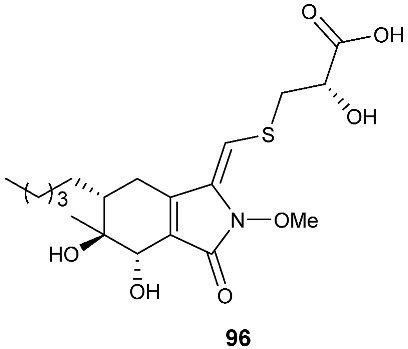	*Paraphaeosphaeria neglecta* FT462	*Lycopodiella cernua*	Antibacterial (**94**) Inhibiting NF-kB (**94**), iNOS (94 and **95**)	[36]
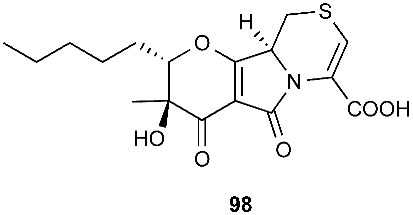	*Paraphaeosphaeria neglecta* FT462	*Lycopodiella cernua* (L.) Pic		[37]
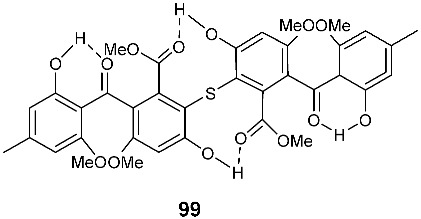	*Guignardia* sp. IFB-E028	*Hopea hainanensis*	Cytotoxic Antimicrobial	[38]
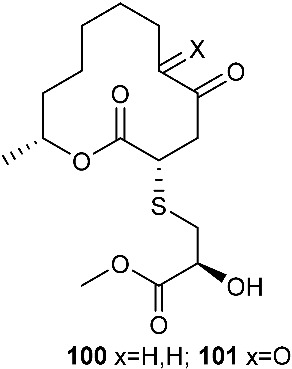 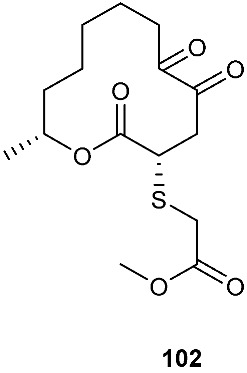 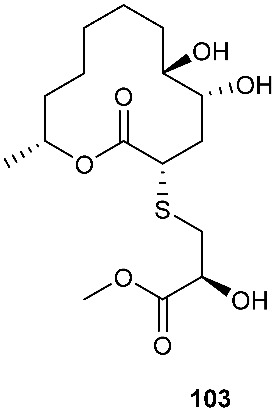	*Cladosporium cladosporioides* MA-299	*Bruguiera gymnorrhiza*	Antimicrobial	[39]
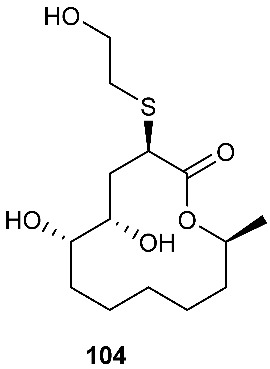	*Cladosporium* sp. SCNU-F0001	Mangrove plant		[40]
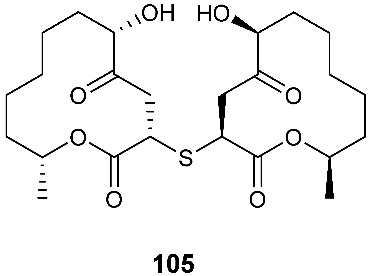 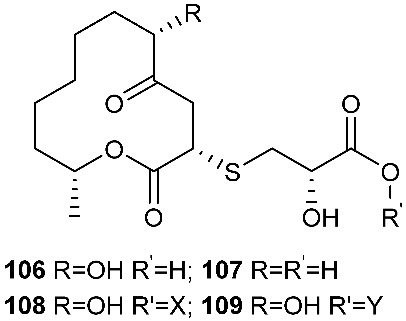	*Cladosporium oxysporum*	Root of *Avicennia marina (Forssk.) Vierh. (Acanthaceae)*	Antimicrobial	[41]
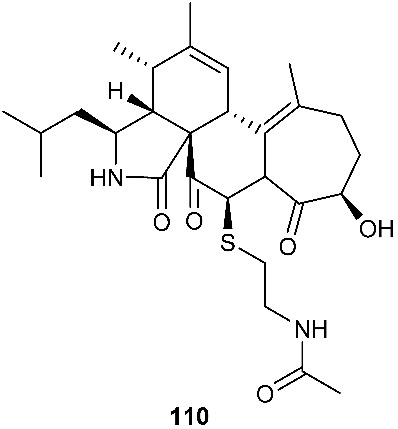 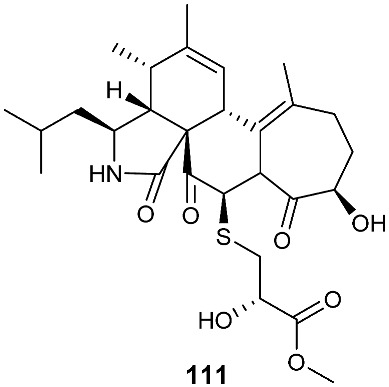	*Aspergillus micronesiensis*	*Phyllanthus glaucus*	Cytotoxic Antibacteria	[42]
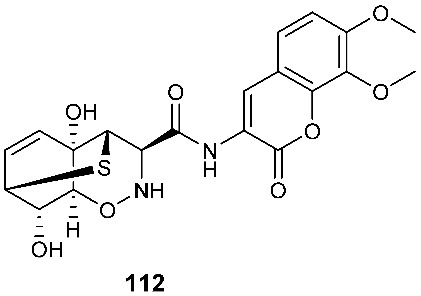	*Trichoderma harzianum* D13	Root of mangrove plant *Excoecaria agallocha Linn*		[43]
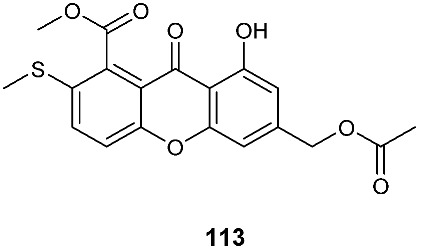 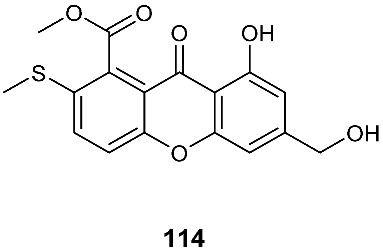	*Aspergillus sydowii*	Livewort *Scapania ciliata S. Lac*		[44]
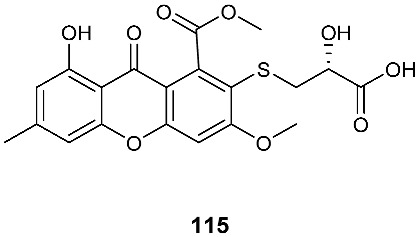 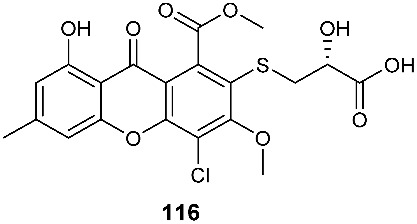	*Pseudopestalotiopsis theae*	Leaves of *Caloncoba welwitschii*		[45]
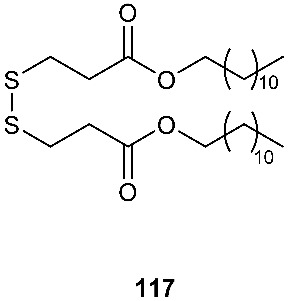	*Sphaceloma* sp. LN-15	Leaves of *Melia azedarach* L.		[46]
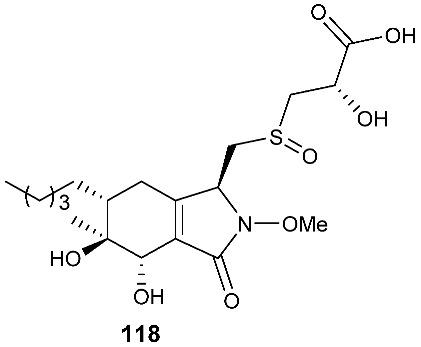	*Paraphaeosphaeria neglecta* FT462	*Lycopodiella cernua*		[36]
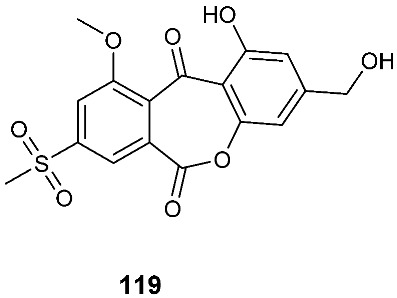	*Neosartorya udagawae* HDN13-313	Root of the mangrove plant *Aricennia marina*	Decreasing the lipid accumulation elicited by oleic acid	[47]
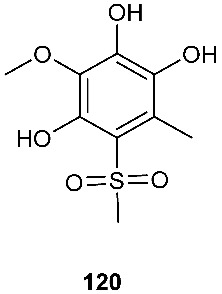	*Neosartorya udagawae* HDN13-313	Root of the mangrove plant *Avicennia marina*		[47]
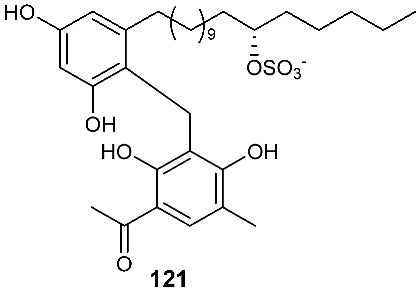 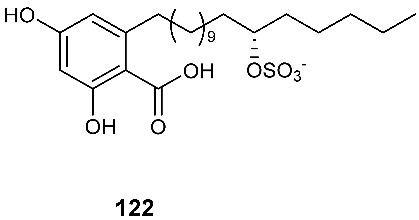	*Penicillium crustosum* PRB-2 and *Xylaria* sp. HDN13-249.	Root of *Sonneratia caseolaris*	Antibacterial	[48]
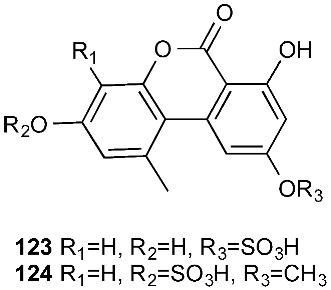	*Alternaria* sp.	*Polygonum senegalense Meisn*. (Polygonaceae)	Cytotoxic (**123**) Inhibiting protein kinases (**123**)	[49]
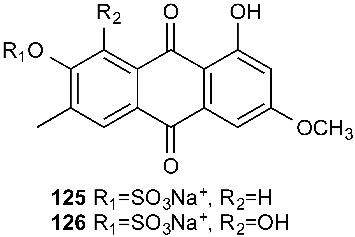	*Ampelomyces* sp.	*Urospermum picroides*		[50]
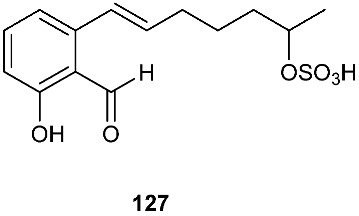	*Pestalotiopsis* sp. AcBC2	*Aegiceras corniculatum*		[51]
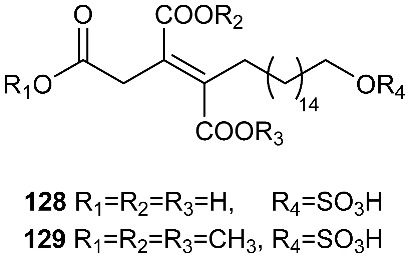	MF6046	Surface-sterilized leaves of *Berberis oregana* (Berberidaceae)	Inhibiting FPTase (**128**)	[52]
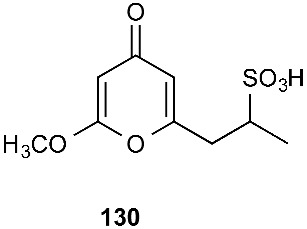	*Fusarium* sp. (CTGU-ZL-34).	*Davidia involucrata*	Cytotoxic	[53]
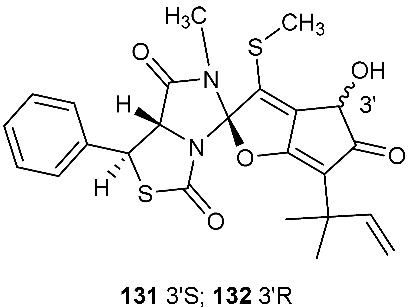	*Pestalotiopsis* sp. HS30	*Isodon xerophilus*	Antitumor	[54]
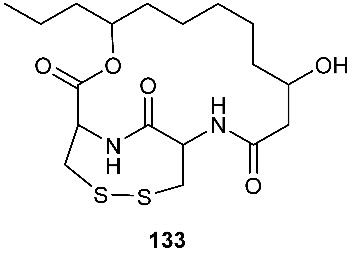	*Phomopsis glabrae*	Leaves of *Pongamia pinnata* (family Fabaceae)	Anticancer	[55]
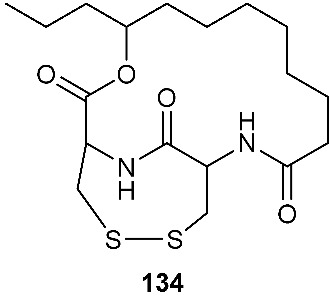	*Ascochyta* sp. AJ 117309	Raw leaf of *Taxus cuspidata var. nana Rehd*	Cytotoxic	[56]
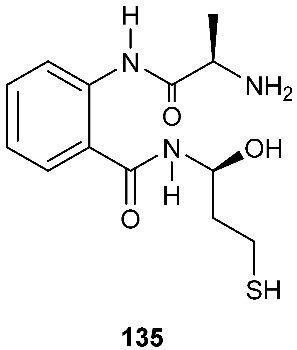	*Fusarium chlamydosporium*	Leaves of *Anvillea garcinia* (Burm.f.) DC. (Asteraceae)	Cytotoxic Antimicrobial	[57]
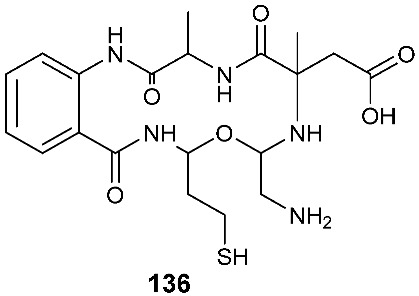	*Fusarium chlamydosporium*	*Anvillea garcinii* (Burm.f.) DC. leaves	Antibacterial Antifungal Cytotoxic	[58]
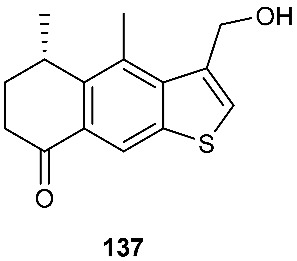	*Leptosphaeria* sp. XL026	*Panax notoginseng*	Antifungal Antibacterial	[59]
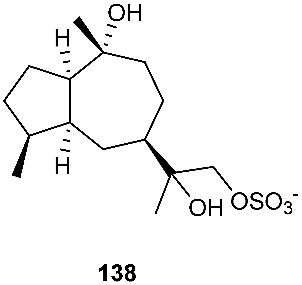 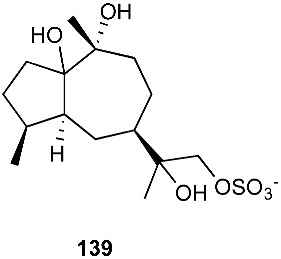	S49	Bark of *Cephalotaxus hainanensis* tree		[60]
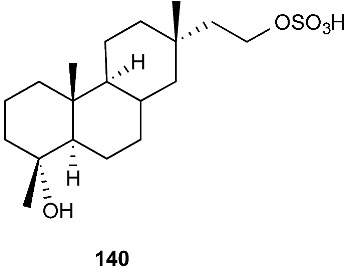 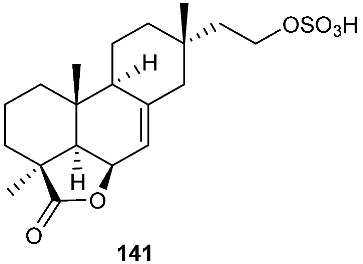	*Xylaria* sp. YM 311647	*Azadirachta indica*	Antifungal	[61]
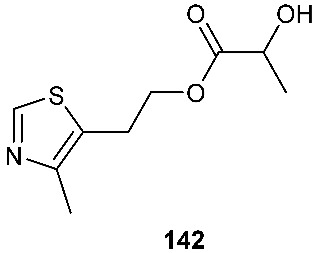	*Colletotrichum gloeosporioides* A12	*Aquilaria sinensis*		[62]
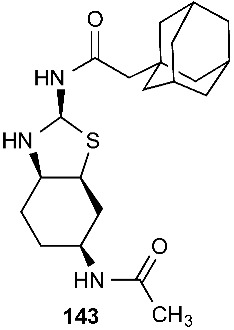	*Emericella Sp*	*Azadirachta indica*	Anticandidal	[63]

## Data Availability

Not applicable.
